# Immediate Consequences of a Spinal Cord Injury During Development: Unique Insights From Ex Vivo Models

**DOI:** 10.1155/np/7460038

**Published:** 2026-04-17

**Authors:** Mariia E. Ermolaeva, Atiyeh Mohammadshirazi, Dimitry Sayenko, Giuliano Taccola

**Affiliations:** ^1^ Neuroscience Department, International School for Advanced Studies (SISSA), Trieste, Italy, sissa.it; ^2^ Applied Neurophysiology and Neuropharmacology Lab, Istituto di Medicina Fisica e Riabilitazione (IMFR), Udine, Italy; ^3^ Department of Neurosurgery, Center for Translational Neural Prosthetics and Interfaces, Houston Methodist Research Institute, Houston, Texas, USA, houstonmethodist.org; ^4^ Department of Neurosurgery, Center for Neuroregeneration, Houston Methodist Research Institute, Houston, Texas, USA, houstonmethodist.org

**Keywords:** ex vivo models, injury potentials, pediatric lesions, physical trauma, remote damage, spinal shock, spontaneous recovery

## Abstract

Over the past 40 years, increasing demand for spinal cord injury (SCI) repair strategies has driven extensive research, yet critical recovery mechanisms remain poorly understood. Key gaps include the temporary loss of spinal reflexes during spinal shock and the dynamics of “injury potentials,” which spread rapidly from the impact site, similar to cortical spreading depression (CSD). While traditionally spinal shock has been viewed as unavoidable, targeted interventions could potentially mitigate SCI pathology and improve recovery. Additionally, immediate changes in brain circuitry post‐SCI remain debated, with limited markers for assessing early neuronal and glial damage. Early supraspinal biomarkers, including neuron‐specific enolase (NSE), S‐100β, and microRNAs, may further refine injury severity assessments. The potential for spontaneous spinal circuit repair is often underestimated, yet molecular evidence suggests preserved interneuronal networks may support functional reconnections. Pediatric SCIs show superior self‐repair, highlighting unique plasticity mechanisms that could be leveraged for therapeutic benefit. While in vivo models mimic human pathology, ex vivo neonatal rodent models allow continuous electrophysiological recordings of spontaneous and evoked neuronal activity during SCI, revealing how lumbar locomotor circuits integrate afferent input post‐injury. Using an ex vivo neonatal SCI model, we demonstrate real‐time network changes in the brain and spinal cord. Our model enables modulation of the extracellular ionic environment and afferent stimulation. By integrating ex vivo models, molecular biomarkers, and insights from early developmental stages, we can uncover novel mechanisms of an acute SCI or refine neuromodulatory strategies to promote recovery of functions.

## 1. An Overview on Traumatic SCIs

A spinal cord injury (SCI) is a neurological condition resulting from a damage to the spinal cord caused by either traumatic events, such as falls, motor vehicle accidents, and acts of violence; or non‐traumatic factors, including tumors, degenerative diseases, vascular disorders, infections, toxins, or congenital abnormalities [1, 2]. However, the majority of SCI cases are trauma‐related, with an estimated global prevalence exceeding 15 million individuals [2]. A traumatic SCI can be viewed as a degenerative pathology of the entire central nervous system (CNS) triggered by an initial impact to the cord, from which the damage spreads until it is finally halted by the formation of a glial scar that encapsulates the main source of endogenous toxic elements at the injury site, such as cellular calcium ions, excitatory amino acids, reactive oxygen species, and mediators of inflammation. As a result, since mature mammalian species have a poor ability to regenerate or repair their CNS after a traumatic insult, an SCI often causes permanent sensory, motor and autonomic deficits over the parts of the body innervated by spinal neurons located below the level of injury. Hence, SCI brings to a life‐long paralysis that is likely worsened by additional functional deficits and complications, such as autonomic dysreflexia, spasticity, and neuropathic pain, all of which significantly impact the quality of life and may lead to secondary health deterioration over time [3]. To date, paralysis cannot be cured, and physical rehabilitation mainly aims at strengthening able muscles to compensate for the loss of volitional motor control and at facilitating activity‐dependent plasticity with moderate yet promising results [4, 5]. Mobility aids, such as wheelchairs and crutches, support the independent performance of daily tasks, and are now being improved by advanced neuroprosthesis, such as exoskeletons [6] and brain machine interfaces [7], which, however, have added only minor functional advantages to date.

Some inconsistencies in existing literature become evident while trying to define the timeline of “early” changes after SCI. Most researchers agree on the subdivision of an SCI into primary and secondary injury. The term primary injury refers to an initial traumatic damage, identified as the immediate vertebral column disruption or dislocation, and the related mechanical forces acting on the spinal cord. The arbitrary time window of a primary injury lies within the first 2 h following the lesion, when abrupt cellular death and axonal disruption happen [1, 8]. After a few hours from the initial impact, a cascade of pathological processes, collectively termed secondary injury, develops. This phase exacerbates spinal tissue damage with further cellular death, amplified to neighboring segments through a plethora of pathological changes: intra‐ and extracellular ionic deregulation, excitotoxicity, edema, ischemia, and inflammatory response, to name a few [1, 9].

In this review, we will adapt the clinically justified subdivision of injury into immediate, acute, and sub‐acute phases [10] and focus on the immediate consequences of SCI that contribute to primary injury at its earliest stage. Moreover, while numerous studies have addressed secondary processes such as inflammation, gliosis, and long‐term tissue remodeling, our focus is deliberately restricted to immediate cellular and network‐level alterations occurring within milliseconds to hours post‐injury, as we aim to provide a precise and mechanistic understanding of the initial post‐injury dynamics.

## 2. Immediate Consequences of an SCI

### 2.1. The Spinal Shock

Despite the growing body of evidence on the pathophysiology of SCI, many events occurring during a physical trauma to the spinal cord remain unexplored. Indeed, it remains unclear to what extent the initial insult to the spinal tissue determines the magnitude of the subsequent secondary damage, which eventually culminates in neuronal death and functional deficits [9, 11–13]. Clinically, the term spinal shock designates the immediate post‑injury phase in which almost all reflex activity below the level of a SCI is transiently abolished or markedly depressed. This temporary suppression of spinal reflexes constitutes a well‑established initial consequence of SCI [14]. The term “spinal shock” was first introduced by Hall [15] in 1840. The classical physiological definition dates back to Sherrington’s “Address on the spinal animal,” where he demonstrated that the abolition of reflexes after acute transection is not due to destruction of reflex pathways but to a temporary depression of spinal excitability following loss of descending inputs [16]. Depending on the reflex being considered for reappearance, spinal shock can last from days to weeks. Sometimes, spinal shock does not last more than 20–60 min if monitoring the recovery of the delayed plantar response, which usually anticipates the reappearance of other reflexes, as cutaneous, deep tendon, and bladder ones [17, 18]. On the other hand, the full recovery of tibial H‐reflex after SCI can take up to 3–4 weeks in rats, 4–6 weeks in cats, and 12–16 weeks in humans [18, 19].

Although the spinal shock has been long known [20–22], hypotheses regarding its underlying mechanisms and progression remain only scattered [18]. A wider literature on neuronal pathophysiology after a traumatic brain injury (TBI) suggests that the immediate mechanical forces leading to the damage of cellular structures are of fundamental importance to understand post‐traumatic pathology [23, 24]. We suggest that this might be true also for SCI. Interestingly, the first pathological responses to a TBI are membrane disruption and the loss of ionic homeostasis, which lead to a dysfunction of axonal conduction [25, 26]. Analogously, during spinal shock, the transient disappearance of reflexes has been mainly attributed to the increased efflux of potassium from neural cells at the injury site, which hinders axonal conduction in the white matter [27–30]. In addition, both the sudden interruption of tonic input from descending fibers, and the increased segmental presynaptic inhibition have been reported to contribute to reflex suppression [27, 28].

In clinics, spinal shock is managed as a transient and inevitable phenomenon that currently receives no specific treatment beyond standard supportive care, such as hemodynamics and respiratory stabilization, and surgical decompression [31–34]. However, providing targeted strategies to reduce the magnitude of spinal shock might mitigate SCI pathophysiology and improve the fate of functional recovery [35, 36].

### 2.2. The Injury Potential

**Figure 1:**
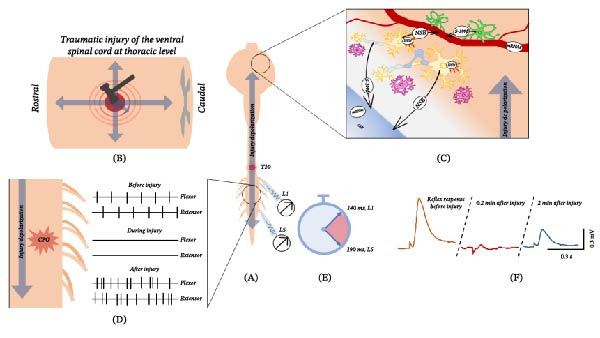
Omnidirectional propagation of a depolarizing wave throughout the CNS following traumatic spinal cord injury. (A) Schematic representation of the whole CNS preparation isolated from neonatal pups. (B) Cartoon illustrating the spread of the depolarizing wave (gray arrows) triggered by a traumatic thoracic impact delivered to the ventral spinal cord. The depolarization propagates in both rostro‑caudal and dorso‑ventral directions, with maximal amplitude near the lesion epicenter and progressive attenuation with increasing distance. (C) Remote effects of SCI on supraspinal structures. Following injury, neurons and glial cells release proteins (e.g., S100β, NSE), microRNAs, and signaling molecules (e.g., HCN channels) into the extracellular space; these become detectable in blood, serum, or CSF and rapidly deviate from baseline levels, highlighting their potential as early biomarkers of brain involvement after SCI. (D) Schematic representation of disturbances in fictive locomotor output during and after the traumatic event, illustrating disruption and disorganization of locomotor patterns. (E) Time course of the caudal spread of the depolarization wave from thoracic to lumbar spinal segments. Recordings from lumbar ventral roots L1 and L5 demonstrate the transient nature of the injury‑evoked depolarization, with onset latencies of ~140 and 190 ms, respectively. (F) Effects of traumatic injury on lumbar ventral root responses evoked by electrical stimulation of dorsal roots (original data). The reduced response amplitude mirrors the depression of spinal reflexes characteristic of spinal shock observed clinically.

Experimentally, the course of a spinal shock matches the massive and transient depolarization of the whole spinal cord, named “injury potential,” which immediately follows the impact and then spreads both rostrally and caudally from the lesion site (Figure 1). At the injury site, several other mechanisms contribute to the depolarization of neurons. First, mechanical membrane disruption results in the outflux of cations. Uncontrolled potassium (K^+^) efflux results in extracellular K^+^ buildup. Increased sodium (Na^+^) fluxes occur through dysfunctional channels, reversed activity of Na^+^/Ca^2+^ exchangers and failure of Na^+^/K^+^ ATPase due to ATP deficiency. These mechanisms lead to the disruption of the extracellular ionic balance and to the abnormal cations uptake by surrounding cells [29, 37–40]. Secondly, a critical determinant of neuronal excitability after SCI is the regulation of intracellular chloride concentration, which is primarily controlled by the opposing actions of the chloride co‐transporters, KCC2 and NKCC1. Following SCI, a rapid downregulation of KCC2 in neurons below and around the lesion site is accompanied by an upregulation or functional dominance of NKCC1. This shift drives the chloride equilibrium potential (*E*_Cl_) toward more depolarized values, thereby converting normally inhibitory GABAergic and glycinergic transmission into less effective or even excitatory synaptic actions. Such alterations have been documented both in adult injury models [41, 42] and in neonatal rodents, where baseline KCC2 expression is naturally low and the system is therefore particularly vulnerable to perturbation [43, 44]. This compromised chloride homeostasis contributes to enhanced motoneuron excitability, aberrant network synchronization, and impaired integration of sensory inputs after SCI. Moreover, dysregulation of KCC2/NKCC1 balance is a well‐established mechanism underlying neuropathic pain, as shown by Coull et al. [45], who demonstrated that reduced KCC2 expression in dorsal horn neurons leads to pathological GABA‐mediated depolarization and mechanical allodynia. Further, an excessive concentration of excitatory neurotransmitters in the biophase, glutamate (Glu) in particular, sustains the depolarized state of neurons. Extracellular glutamate accumulation is sustained both by the release of Glu from local presynaptic and sensory neurons, and from primary afferent fibers, as well as by the reduced clearance of Glu from the synaptic cleft through glutamate transporters [46, 47]. The excess Glu in turn binds to ionotropic NMDA and AMPA receptors of neighboring neurons, causing prolonged receptor activation and leading to a rapid influx of Ca^2+^ ions [48]. The increased availability of Ca^2^ contributes to the alteration of neuronal excitability through a SNARE‐mediated vesicular release, and frequently initiates apoptotic signaling cascades culminating in neuronal death [49]. Furthermore, immediate massive depolarization caused by receptor overactivation transiently drives voltage‐gated Na^+^ channels from the closed to the inactivated state. Since Na^+^ channels must repolarize to return from the inactivated to the closed state before reopening, this leads to a temporary inability to generate action potentials, a phenomenon termed “electrical silence” [50]. Additionally, the sustained opening of Glu receptor channels and other ion channels increases membrane conductance, effectively reducing the neuronal input resistance. This reduction in input resistance shunts incoming synaptic currents, further dampening neuronal excitability [51, 52].

Following the initial neuronal depolarization, a wave of spreading depolarization (SD) can arise and propagate through spinal tissue or brain. For instance, in the cerebral cortex, this phenomenon is known as cortical spreading depression (CSD; [53, 54]), defined as a slowly propagating (2–5 mm/min in humans; [55]) wave of near‐complete depolarization of neurons and glia that disrupts normal electrochemical gradients [37, 56, 57]. CSD is triggered, among ischemia and other causes [58], by migraine [59] and TBIs [60]. The SD disrupts electrochemical gradients [37, 56, 57] and causes extracellular K^+^ accumulation, that leads to a massive release of glutamate from neurons, astrocytes, microglia, and vascular smooth muscle cells [61–63]. In neurons, this results in swelling and distortion of dendritic spines [50], which in turn impairs synaptic function and contributes to decreased intrinsic excitability. Astrocytes and microglia also depolarize and their depolarization alters ion and neurotransmitter homeostasis, exacerbating excitotoxic conditions. For instance in astrocytes, depolarization impairs K^+^ buffering and reduces Glu uptake through excitatory amino acid transporters (EAATs), leading to elevated extracellular K^+^ and Glu levels [64]. Microglia, on the other hand, promotes the release of pro‐inflammatory cytokines and reactive oxygen species [65, 66]. These changes amplify excitotoxic stress and neuronal vulnerability. Depolarization of vascular smooth muscle cells causes vasoconstriction, affecting local blood flow and metabolic supply [67]. Similar SD‐like waves and temporary rise in extracellular K^+^ concentrations occur in the spinal cord after SCI, as demonstrated in rodent and amphibian models [68, 69]. These spinal SD‐like waves propagate at ~10–15 mm/min and are initiated by abrupt increases in extracellular K^+^ concentrations. During these waves, electrically evoked potentials are transiently suppressed and recover only after about 20 min, suggesting a role in the temporary loss of reflexes seen in spinal shock [69]. When trauma affects the brain, CSD originating at the impact site can propagate to upper spinal segments, reducing excitability in motor pools and indicating interconnected cortical and spinal SDs [69].

## 3. Acute Consequences of an SCI on Remote Regions of the CNS

Although extensive, the current literature on the acute and subacute consequences of SCI provides limited evidence about the immediate electrophysiological changes occurring in brain circuits following SCI, but this is likely due to experimental constraints. Acute changes in the brain after SCI remain a subject of debate, with findings ranging from no cellular loss [70] to massive retrograde neurodegeneration [71, 72] with some evidence suggesting more complex interactions [73, 74]. Interestingly, a detailed study on sub‐acute SCI in mice reported cognitive deficits and depressive‐like behaviors, associated with a reactive microglia and neuronal loss both in the hippocampus and in the cerebral cortex [74]. However, no significant neuronal death was detected in the brain during the first 2 weeks after SCI, suggesting that the behavioral changes were secondary to late inflammatory processes.

Recent studies using brain‐spine interfaces, neuromodulation, and targeted rehabilitation protocols provided significant progress in the role of supraspinal pathways in modulating spinal plasticity and promoting motor recovery after SCI [75–77]. However, these efforts have primarily focused on how descending cortical or brainstem inputs shape spinal circuit reorganization and motor output. For instance, Bonizzato and colleagues demonstrated that closed‐loop brain stimulation can enhance locomotor recovery by reinforcing corticospinal transmission. However, whether the initial depolarization events and ionic disruptions that occur in the spinal cord after injury can propagate retrogradely and impact supraspinal structures has not been explored sufficiently. SCI has been shown to initiate pathophysiological cascades that are not restricted to the injury site. For instance, SCI can alter synaptic transmission in supraspinal regions such as the sensorimotor cortex and thalamus by disrupting the balance between excitatory (glutamatergic) and inhibitory (GABAergic) signaling, leading to cortical hyperexcitability and even irreversible axonal dieback in descending motor pathways [78]. Beyond structural degeneration, SCI triggers maladaptive central responses that can give rise to central sensitization, a state in which neurons in the spinal dorsal horn and supraspinal centers become hyperresponsive [79]. This involves increased excitatory neurotransmitter release, altered ion channel expression, reduced inhibitory tone, and microglial and astrocytic activation [46, 80–83]. These mechanisms contribute to exacerbating pain signaling and potentially leading to hypersensitivity, neuropathic pain, and spasticity [84]. These changes are not immediate or acute, as they require several hours or even days to be consistently detected and assessed. However, scattered electrophysiological studies describe functional changes occurring in the cortical circuitry at least 30 min after the spinal cord transection in rodents [73, 85]. Changes in evoked cortical responses, represented by local field potentials, suggest that a functional reorganization takes place in the primary somatosensory cortex after SCI, although this data is not, per se, sufficient to confirm that the immediate changes in cortical neuron properties are caused by the disconnection from the spinal cord. In a more recent study, the same group detected the immediate alterations of the pattern of sensorimotor cortex oscillations by performing continuous extracellular recordings during a complete spinal transection [86]. Studies in anesthetized rats indicate how the characteristic slow‐wave activity across all layers of the sensorimotor cortex is disrupted by a short period of sustained depolarization occurring milliseconds after SCI, which is followed by a restoration of slow‐wave oscillations. Later on, some layer‐specific changes appear during extracellular registrations of both spontaneous and evoked activities, highlighting a net decrease in spontaneous activity and a higher network excitability. Interpretation of this data may be biased by the potential masking effect of anesthesia over some transient and subtle changes in neuronal activity during spinal cord transection.

## 4. Early Biomarkers in the Brain Might Trace the Extent of an SCI

Quantifying biomarkers expressed in the brain immediately following SCI may help refining current assessments of lesion severity and clinical prognosis. While not yet routinely used, such measures could enhance our understanding of individual variability in recovery potential. Certainly, an extensive body of evidence exists regarding the biomarkers in SCI and their diagnostic role [87–89]. However, they do not address what biomarkers could reflect SCI in the brain. Some markers have been suggested to assess neuronal and glial damage during the acute phase of SCI (Figure 1b). For instance, the level of neuron‐specific enolase (NSE), which is a cytoplasmic glycolytic enzyme localized in neurons and neuroendocrine cells, significantly increases 55 min after thoracoabdominal cross‐clamping in dog CSF [90] and after 2 h in SCI rat model’s blood serum and CSF [91]. S‐100β, a calcium‐binding protein localized in the cytoplasm of astroglia and Schwann cells, supports neurite outgrowth and provides protection against oxidative stress [92], as well as representing an early biomarker of lesion progression after brain or SCI [93, 94]. Notably, S‐100β expression increases in serum and SCF of SCI rats after 2 h, reaches the peak at 6 h, and decreases at 12 h and even further at 24 h after SCI [91]. Moreover, S‐100β levels are correlated with injury severity, as confirmed by an ex vivo chemical model of SCI, where extracellular levels of S100β in the spinal cord raised after lesions evoked by increasing concentration of kainate (Figure 1d; [93]). As reviewed by Rodrigues et al. [95], another potential marker for SCI in the brain is microRNAs. These molecules can be obtained with a minimally invasive procedure, for example blood sampling, and are highly specific to the tissue collected, reflecting acute changes in the cellular machinery. Mouse models of SCI indicate that levels of microRNAs 124a and 223, extracted directly from injured spinal cords, are altered after SCI in a time‐dependent manner, over a period ranging between 12 h and 7 days. On the other hand, no research appears to have explored levels of cortical microRNAs after SCI, such as miR‐93, miR‐191, and miR‐499, which have been proposed as markers for TBI. Indeed, their levels in the serum rise within 48 h after TBI, which is clinically considered the acute phase. Additional data suggests that other potentially predictive changes in microRNA levels can be detected even earlier [96], such as the levels of serum miR‐425‐5p and miR‐21, which can be assessed 4–12 h after TBI and are strongly correlated with the outcome [97].

A recent study on depolarization and hyperexcitability in the cerebral cortex after axotomy suggests the involvement of hyperpolarization cyclic nucleotide (HCN) ‐gated channels in the control of neuronal hyperexcitability [98]. However, as Najemet al. [99] emphasized, the timing of serum sampling relative to the injury is crucial for accurately assessing its severity. Altogether, while several candidate biomarkers show potential for reflecting SCI‐related changes in the brain, further research is needed to validate their temporal profiles, specificity, and prognostic value in clinical settings.

## 5. Spontaneous Recoveries From SCI

Anecdotal observations described spontaneous functional recoveries in persons with SCI [100], typically plateauing around 16 weeks post injury, with remarkable functional gains especially in case of milder injuries [101]. Notably, SCIs due to the lateral hemisection of the spinal cord, lead to a temporary paralysis, but are often followed by a spontaneous recovery of walking abilities both in mice and in humans [102–106]. Spontaneous recoveries keep challenging the consolidated view about the degenerative pathophysiological mechanisms of an SCI and the negligible residual potential of the cord to repair spinal circuits. Interestingly, several clinical trials on therapeutic strategies for SCI have documented spontaneous functional recoveries in subjects from placebo cohorts [101, 107, 108]. The likelihood of a neurological recovery greatly depends on the retained neurological function below the level of injury, meaning that a better‐preserved function below lesion predicts a better outcome. One clinically relevant feature is the zone of partial preservation (ZPP), sublesional areas where motor or sensory function is retained even in individuals classified as having complete injuries. Often, significant spontaneous motor recoveries within the ZPP are attributed to CNS plasticity rather than actual axonal regeneration [109]. However, some parameters of ZPP, such as extent, precise localization and borders, vary among individuals and are difficult to determine. A consistent and detailed characterization of the ZPP across subjects would offer multiple therapeutic advantages by improving outcome prediction and guiding of personalized interventions and rehabilitation [7, 110, 111].

Spontaneous recoveries in supraspinal regions of the CNS have been studied extensively in animal models. In rodents, the motor cortex undergoes major structural changes after SCI, including atrophy and degeneration of corticospinal projections [112, 113], but also corticospinal sprouting [114, 115] and, in some cases, partial reestablishment of corticospinal connectivity [114, 116]. For example, in mice with cervical SCI, the motor cortex can restore some output to the limbs, enabling partial recovery of skilled locomotion [117]. Chemogenetic silencing of spared CST neurons in these mice significantly increased paw placement errors during the ladder task, demonstrating that uninjured CST neurons are essential for spontaneous recovery. Moreover, species differences significantly shape recovery. Mice show greater corticospinal plasticity and faster restoration of function than rats, especially after incomplete lesions. After cervical SCI, mice can partially re‐establish CST output to limbs [117], whereas rats exhibit more limited spontaneous CST remodeling [118]. These differences arise from species‐dependent sprouting capacity, inflammation profiles, and astrocytic responses. Recovery also depends strongly on lesion type and location: thoracic contusions generally produce modest spontaneous recovery, whereas cervical hemisections or dorsal hemisections elicit more robust compensatory rearrangements. In contrast, lumbar injuries show limited spontaneous reconnection because long descending fibers are relatively sparse at these segments. Nonetheless, intrinsic lumbar circuits retain the ability to undergo plasticity and can still generate locomotor rhythmogenesis when appropriately engaged [119].

Neuromodulatory systems, including serotonin, noradrenaline, and glutamate, play an essential role in shaping the excitability of central pattern generator (CPG) circuits. Serotonin and noradrenaline robustly facilitate rhythmogenesis and motoneuron excitability, enabling the lumbar cord to generate locomotor‐like activity even with greatly reduced descending control [120–122]. Glutamatergic transmission provides the core excitatory drive required for CPG oscillations [123, 124]. After SCI, these neuromodulators can help stabilize network dynamics, enhance responsiveness to afferent input, and support endogenous locomotor function in sublesional circuits, contributing to spontaneous recovery of stepping [125–127].

Endogenous repair processes also contribute to spontaneous improvement after incomplete SCI. Early recovery during the first days to weeks is shaped by metabolic and structural compensations in spared tissue [128, 129]. Demyelination near the lesion can be followed by spontaneous partial remyelination mediated by infiltrating Schwann cells or activated oligodendrocyte progenitor cells [130]. At the circuit level, functional improvement is supported by sprouting of axonal branches, dendritic reorganization, and strengthening of pre‐existing synapses in surviving pathways [1, 128].

Advanced genetic and transcriptomic tools have enabled high‐resolution profiling of cellular responses to SCI. Russ et al. [131] compiled a comprehensive atlas of spinal cord cell types using single‐cell RNA sequencing, providing a foundation for mechanistic studies. Building on this resource, Matson et al. [132] mapped molecular changes in cell populations after moderate thoracic contusion in mice, analyzing both the lesion site and the sublesional lumbar cord. These studies have shown gene‐expression response within 1 day post‐injury in microglia, while neurons displayed transcriptional signatures of cellular stress and plasticity only after 1 week. Notably, transcriptomic analysis identified a cluster of cells expressing regeneration‐associated genes (RAGs), including Atf3 and Sprr1a., and elevated Sprr1a expression was confirmed in vivo in neurons located near the lesion. These cells were likely spared spinocerebellar neurons and Shox2 (V2d) interneurons, potentially capable of spontaneous remodeling although definitive anatomical evidence of axonal regrowth is still lacking. The identification of RAG‐positive neurons aligns with the concept that neuromodulation can promote functional reconnections through preserved interneuronal pathways [133–135], promising for enhancing volitional motor recovery.

## 6. Traumatic Injuries to the Immature Spinal Tissue

Pediatric SCIs account for 1%–10% of all SCIs [136] and show a greater likelihood of a spontaneous functional recovery compared to adults [137, 138]. Thus, investigating traumatic injuries during development is compelling to clarify the unique pathophysiology of neonatal SCIs and might also reveal the mechanisms behind the greater chances of recovery. The developing spinal cord experiences distinct direct and indirect forces and are exposed to different types of traumas than adults [139, 140]. However, comparing pediatric SCIs with those in adults may help reveal the mechanisms underlying the greater recovery often seen in pediatric cases and potentially harness these mechanisms to benefit all individuals with SCI [141]. Interestingly, pediatric SCIs experience a shorter spinal shock, likely due to the immaturity of their descending tracts. Moreover, pediatric SCI compromises tonic inhibition from the brain over spinal pathways in a milder manner compared to adults, with a more contained depression of spinal network activity during shock [142].

Compared to adults, neonatal mammals exhibit a superior ability to self‐repair [143, 144], particularly as for sprouting of growing neurites [145–147]. Moreover, it has been shown that rats that undergo a spinal transection at P14 exhibit most of the fibers intact 5 weeks later, including rubrospinal, vestibulospinal, and reticulospinal tracts, demonstrating axonal regeneration [148]. Similarly, in 1‐day‐old cats, a spinal cord hemisection induced corticospinal projections to circumvent the lesion to restore motor functions: a phenomenon that cannot be observed in adults [149]. Apparently, development alters the acute pathophysiology of an SCI by worsening neuroinflammatory responses [150, 151]. Circuitry maturation is also associated with a reduced trophic factor and cytokine secretion, an impaired axon growth, and a diminished recruitment of macrophages at the lesion site [152]. Both experimental and clinical studies in animals and humans confirm that younger individuals exhibit greater neuronal plasticity, including the ability to reorganize neural pathways and promote axonal sprouting, leading to improved neurological outcomes following SCI [138, 153–155]. Further investigation is needed to elucidate the mechanisms behind the pathophysiology of neonatal SCIs and the enhanced recovery in children.

## 7. Current Rodent Models of SCI: Strengths and Weaknesses

Since its introduction in 1911 by Allen [156], the original weight‐drop impactor underwent various modifications that have been implemented and validated for a widespread use in adult rodents [157–159]. In vivo models of rodent SCI are widely adopted for their affordability and resemblance of human pathology. Another advantage is that SCI progresses faster in rodents, making 4 weeks after injury sufficient to reach the sub‐acute phase, whereas larger mammals would require several months. Currently, rat is the most used model for behavioral assessments of motor deficit after SCI, as it is functionally, electrophysiologically, and morphologically closer to humans. In particular, large fluid‐filled cystic cavities surrounded by glial scar tissue, closely resembling the human pathology, are typically formed at the injury site in rats [160, 161]. In contrast, such cavities are uncommon in mice following experimental SCI. Instead, the lesion core becomes filled with fibrotic scar tissue [162], with only occasional microcystic cavitation reported under specific injury protocols [163]. Moreover, mice are less robust to surgical procedures compared to rats and are phylogenetically farther from humans. Nonetheless, they remain indispensable for SCI research when refined genetic manipulations are required. Larger mammalian models, such as Yucatan minipigs, enable the use of clinically validated technologies and enhance translational relevance in spinal cord research [135, 164]. However, their use entails substantial financial and logistical demands, which currently constrain the feasibility of large‐scale studies. Collectively, rat preclinical models provide the best “cost‐effectiveness” balance for studying SCIs. Despite the significant advantages offered, preclinical rodent models of SCI currently available do not fully allow exploration of spinal shock and immediate transient changes happening in the brain. Indeed, due to ethical concerns, all in vivo models require animals to be completely anesthetized before injury, hence moving away from the unpredictable nature of clinical traumas. Indeed, anesthetics impact the progression of a lesion, as the different drugs used can either worsen hypoxic neuronal injury creating a transient hypotension [165] or, alternatively, act as a neuroprotector [166–168]. Significant neuroprotection, in terms of a mitigated cell death following excitotoxicity and ischemia, is offered by common anesthetics, like barbiturates and isoflurane [167, 169]. In particular, isoflurane delays preconditioning against spinal cord ischemic injury through the release of free radicals, as observed in rabbit models [170], and propofol offers neuroprotection by reducing motoneuron loss against kainate‐induced excitotoxicity in the spinal cord [171]. Similarly, ketamine, acting as a noncompetitive NMDA (N‐methyl‐D‐aspartate) receptor antagonist, has shown strong protective effects against spinal cord ischemia and reperfusion injury and has been effective in preserving antioxidant activity within spinal cord tissues [172].

Additionally, in anesthetized in vivo models, the electrical interference generated by the engine of currently available impactors, covers the low‐amplitude electrical signals recorded from spinal neurons at the time of the physical trauma. As a result, the earliest injury potential recorded in fully anesthetized animals can only occur after at least 4 min from the impact [173], missing all the earliest electrophysiological signs of physical trauma. Moreover, the required electrode repositioning after the trauma prevents accurate assessment of post‐injury changes in spinal cord DC potential. This limitation hinders the ability to track subtle shifts in electrical homeostasis that may reflect early pathophysiological responses. Furthermore, preclinical models of SCI are mainly restricted to inducing dorsal SCIs, as accessing the ventral cord would require highly invasive procedures involving temporary displacement of thoracoabdominal organs. Due to the complexity and risk of such interventions, replicating ventral SCIs, despite their high prevalence in clinical populations [1], remains impractical in vivo experimental settings.

Conversely, ex vivo models, despite lacking blood flow and systemic immune responses and being unsuitable for assessing long‑term recovery, remain valuable tools for investigating the immediate biomechanical consequences of trauma on the CNS. Crucially, this approach eliminates the need for chemical anesthesia during trauma induction, thereby reducing the risk of systemic cardiovascular instability and avoiding anesthesia‐induced network suppression or hypoxic neuronal damage [174–176]. Studies using stretch [177] and shear strain paradigms [178, 179] have advanced our understanding of axonal mechanobiology and membrane deformation in cultured cells and organotypical or acute CNS slices. Various preparations such as spinal cord strips, organoids, and organotypic slice culture in rodents are widely employed to emulate compression [69, 180–182], weight drop [183, 184], transection [185], and chemical injury [186]. In particular, isolated strips of white matter from the spinal cord of adult guinea pigs have been adopted to compare the early axonal responses following both, contusion and transection injuries in vitro [187]. In this preparation, both types of lesions evoke rapid depolarizing potentials that similarly arise within seconds, regardless of the type of insult [187]. This is not surprising considering that, even though contusion and transection arise from distinct pathophysiological mechanisms, they display a remarkably similar pattern of acute progression [188]. Indeed, by 4 days post‑lesion, neither behavioral outcomes nor corticospinal signals differed among the injury models. A distinct divergence in injury progression began to emerge only after 1 week across groups; however, severe contusions continued to exhibit outcomes comparable to complete transection throughout the entire sub‑acute observational period (3 weeks; [188]). Furthermore, highly reproducible ex vivo models of SCI have been developed using transverse or longitudinal cord slices from neonatal rats to dissect both acute and secondary injury mechanisms with controlled experimental precision [189]. Using ex vivo neonatal reduced preparations provides significant advantages for investigating SCI across early developmental stages.

Despite their advantages, current ex vivo models offer only cellular‐level access, at the expense of the physiological complexity inherent in longitudinal propriospinal connections and supraspinal inputs. This significant limitation can be partially addressed by employing more intact CNS explants, provided that the technical challenges of maintaining such structured preparations viable in vitro are overcome. A significant advance in this direction was introduced by Stirling and colleagues, who established an ex vivo en bloc cervical spinal cord preparation and employed two‐photon microscopy to visualize real‐time cellular and subcellular dynamics within white matter, capturing acute axonal injury, axonal retraction, and myelin degeneration, following a precisely targeted, laser‐induced spinal cord lesion [190].

## 8. An Ex Vivo Model to Mimic Calibrated Impacts to Neonatal Spinal Cords

The acute consequences of SCIs during early developmental stages can be explored using neonatal preparations; however, only a limited number of studies have addressed this by adopting samples of immature spinal tissue [150, 151, 191, 192]. Early foundational work by the Vinay group was instrumental in establishing the neonatal spinal cord preparation to study the acute and subacute consequences of SCI [193, 194]. Using in vitro spinal cord preparations from neonatally transected rats, [193] demonstrated that complete transection at the thoracic level produces an immediate disruption of the lumbar locomotor pattern, including impaired coordination and rhythmogenesis, yet these deficits are largely reversible, revealing a remarkable capacity for rapid functional reorganization in immature circuits. Complementary work by Jean‐Xavier et al. [194] showed that inhibitory synaptic inputs onto lumbar motoneurons remain depolarizing for extended periods after neonatal transection due to altered chloride regulation. This persistent excitatory action of GABA/glycine contributes to motoneuron hyperexcitability and provides mechanistic insight into early network dysfunction following SCI.

**Figure 2:**
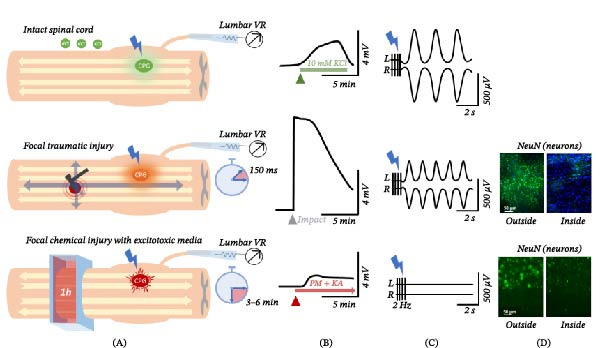
Distinct acute effects of two spinal cord injury models in neonatal rat preparations. (A) Upper panel: Diagrammatic representation of the isolated neonatal rat spinal cord preparation perfused with 10 mM KCl, showing intact and functional locomotor central pattern generators (CPGs) in the lumbar enlargement. Middle panel: A focal traumatic injury to the ventral thoracic spinal cord (T10) is delivered using a high‑precision impactor. Gray arrows illustrate the omnidirectional spread of the injury‑evoked depolarizing wave, which rapidly affects the locomotor CPGs. The depolarization reaches the lumbar cord within 150 ms after impact, as recorded from lumbar ventral roots. Lower panel: Chemical model of secondary injury. A focal chemical insult is induced by a 1‑hour application of a toxic cocktail, mimicking key mediators of secondary damage, restricted between sealed barriers around two spinal segments (T13‐L1). This exposure produces extensive white‑matter damage at the thoracic level, with lower lumbar ventral roots depolarizing within 3–6 min from onset. (B) Schematic signal profiles of KCl‑induced depolarization in the intact preparation (upper panel), traumatic injury–evoked depolarization (middle panel), and chemically induced depolarization (lower panel). Calibration bars indicate the average amplitude and duration for each condition. (C) Schematized traces of fictive locomotion in intact (upper), traumatic (middle), and chemical injury (lower) conditions during dorsal root stimulation with 2 Hz pulse trains. Electrically evoked fictive locomotion is altered following traumatic impact (middle) and abolished after chemical injury (lower). (D) Histological comparison of lesion‑site tissue following traumatic (upper; original data) and chemical (lower; adapted with permission from Taccola et al., 2009, Eur. J. Neurosci.) spinal cord injuries. Sections show regions caudal to the lesion (“outside”) and within the lesion core (“inside”), stained for NeuN‑positive neurons.

To study the immediate consequences of an SCI and avoid any technical constraints and side effects induced by anesthetics, we introduced two standardized and calibrated ex vivo models of SCI using neonatal preparations (Figure 2). A first ex vivo model consisted of spinal cords isolated from neonatal rats (P0–P4), which can remain functional in vitro for up to 48 h. Here, a chemical insult mimicking the secondary damage cascade was focally applied to few thoracic segments (Figure 2a; [195–197]). The study revealed that a chemical lesion evoked a large depolarization (around 1–2 mV) spontaneously recoded from all spinal nerves, which started almost 2–3 min after the first toxic exposure and then propagated to rostral and caudal segments. At the site of lesion, the secondary damage‐like event irreversibly suppressed synaptic transmission evoked by afferent stimulation. However, spinal reflexes beyond the lesioned area were still present, although transitorily halved. Despite the large histological damage to the white matter at the site of injury, as evidenced by a 52% reduction in myelin basic protein and a 66% decrease in mature oligodendrocytes immunostaining [195], remaining connections were sufficient for functional coupling among segments above and below the damage. Moreover, locomotor circuits located in the lumbar enlargement below the level of the injury were transiently impaired, albeit recovering 24 h later if directly activated by neurochemicals. On the other hand, when attempting activation through DR stimulation trains, circuits failed to generate fictive locomotion, showing strong regional decoupling between afferent inputs and locomotor networks. As a matter of fact, even strong DR stimuli lost their ability to reset the cycle of an ongoing locomotor rhythm elicited by neurochemicals.

More recently, a novel layout was devised using the entire CNS [198, 199] isolated from rat pups (P0–P4) with DRGs intact, which underwent a calibrated physical trauma at the tenth thoracic segment using an ad hoc created impactor (Figure 3; [200]). This technique provides unobstructed access to both dorsal and ventral regions of the cord, enabling precise mechanical manipulation. Moreover, the device’s low‐noise design matched the stability of the cord at the impact site, to ensure continuous and stable electrophysiological recordings from multiple ventral roots during and after the impact, without any artifacts corrupting signal acquisition. To our knowledge, no other electrophysiological system currently allows real‑time monitoring of injury potentials in ex vivo CNS tissue at the instant of mechanical impact. This new ex vivo experimental platform also allows to finetune the extracellular ionic environment with very consistent injuries among animals. In addition, multiple segmental spinal reflexes are derived through the precise afferent stimulation of dorsal roots (DR). Using this setup, we quantified the immediate and massive depolarization following a physical insult to the spinal cord, and continuously tracked its propagation both caudally to the lumbar enlargement and rostrally to brain structures (Figure 2b; [200]). The study revealed a massive (5–7 mV of amplitude) and rapid depolarization evoked during the trauma starting only 150–200 ms after the onset of the mechanical compression. Injury potentials then spread centrifugally from the injury site, sustained by an extracellular dysregulation of ions, especially chloride. Notably, both ascending and descending input traveling along the cord underwent a complete functional interruption at the site of the impact, with a net disconnection of spinal networks above and below the injury, which resembled a severe complete SCI (Figure 2b). The transient suppression of all spinal reflexes and their recovery 30 min after the trauma are reminiscent of the spinal shock phase. Moreover, a remote dysfunction of lumbar locomotor networks was observed for up to 3 h, despite the unaffected morphology of lumbar motor pools.

Figure 3Schematic overview of the experimental platform enabling real‑time analysis of acute responses to spinal trauma. (A) The experimental setup combines a low‑noise, high‑precision micro‑impactor mounted on a magnetic base with an ex vivo preparation of the entire central nervous system (CNS), including intact dorsal root ganglia (DRGs), isolated from neonatal rats and continuously superfused with oxygenated physiological solution at 27°C. The micro‑impactor delivers a controlled vertical impact to the ventral surface of the T10 spinal segment. Impact parameters, including depth, velocity, acceleration, and compression duration, are fully programable through dedicated control software, allowing highly reproducible and finely graded traumatic injuries. The platform ensures stable, artifact‑free electrophysiological recordings both during the impact and throughout the immediate post‑injury period. (B) Simultaneous extracellular recordings performed with miniature glass electrodes from cervical and lumbar ventral roots at the moment of the trauma reveal injury‑induced depolarizing potentials. Latency increases with anatomical distance from the lesion site, consistent with the schematic representation on the right illustrating the distance‑dependent spread of the depolarization.

**Figure:**
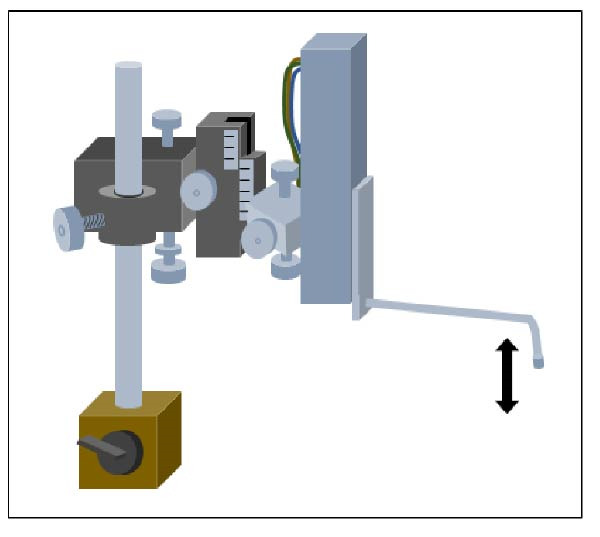
(A)

**Figure:**
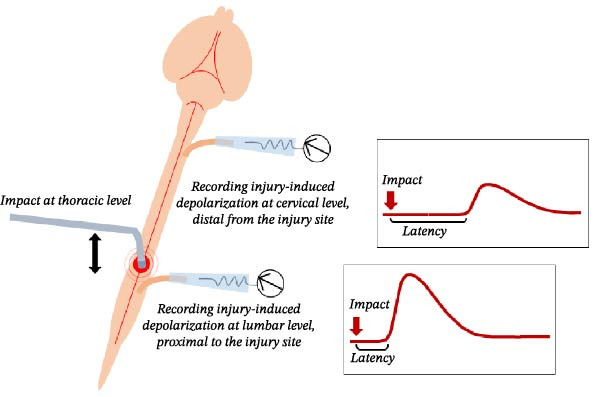
(B)

Our neonatal SCI model, based on the entire ex vivo CNS, provides unique access to the earliest injury‐induced physiological changes across virtually any CNS region, spanning multiple levels of complexity, from systemic to cellular and molecular [94]. Our findings highlight the rapid and region‐specific alterations in cortical astrocyte populations following SCI. Notably, we observed a significant reduction in the density of both S100β^+^ and GS^+^ astrocytes in the primary motor cortex as early as 25 min post‐injury, with no further changes detected at the 2‐h mark. In contrast, astrocyte density in the primary and secondary somatosensory cortices remained unchanged during the initial phase but showed significant alterations 2 h after injury. This spatiotemporal pattern of glial response closely parallels the propagation of SD from the spinal lesion site and may serve as a novel biomarker for assessing lesion severity and predicting functional recovery.

Albeit our ex vivo approach is limited to neonatal tissue due to its stronger survival, some research promotes the idea that adult SCIs are followed by an increased brain plasticity that brings the sensorimotor cortex to a state similar to early development [201, 202]. Moreover, after SCI in adults, the remodeling of spines of cortical neurons that project to the spinal cord indicates an increased plasticity of synaptic connectivity, which is usually observed in the immature CNS [24, 203]. These observations support the feasibility of applying our neonatal ex vivo model to explore any transient changes, even in the brain, in response to SCI.

Although the use of isolated spinal cord preparations to investigate injury mechanisms may appear counterintuitive, given that the procedures required for isolation introduce an initial insult, this concern is mitigated experimentally. The dissection‐related trauma is minimized, and the tissue is subsequently allowed to recover for nearly 2 h prior to the experimental impact. This recovery interval restores metabolic homeostasis and functional responsiveness, as demonstrated in previous studies [199, 200]. Importantly, this period ensures that the experimentally induced lesion is temporally and physiologically distinct from any dissection‐associated damage.

In addition, the time needed for both dissection and following incubation in the recording chamber for a few hours challenges neuronal excitability to a certain extent. Indeed, although the perfusing medium continuously provides an excess of glucose and oxygen to the tissue, even the thin neonatal sample may not equally receive nutrition to all districts of the CNS, likely affecting excitability of distinct areas [204]. However, our results demonstrated optimal functional and histological preservation for up to 4–6 h post‐dissection, with neonatal samples exhibiting greater resilience compared to 1‐week‐old explants [198, 199]. Besides their intrinsic limitations, isolated preparations have been pivotal in deciphering the functional organization of spinal networks [205–207] and still represent a worthwhile benchmark in spinal neurophysiology [79]. The proposed experimental approach allows to further investigate neuronal plasticity during development, even in association with a calibrated injury.

In summary, our platform is not designed to substitute preclinical models used for clinical translation, but rather to serve as a strategic intermediary for testing foundational hypotheses on the immediate impact of mechanical trauma to neural tissue. It offers a rare opportunity to capture the early electrophysiological and cellular events underlying spinal shock, a phenomenon often overlooked in mainstream SCI research. While the use of neonatal tissue limits direct extrapolation to adult pathophysiology, its superior viability in vitro enables high‐resolution analysis and opens a valuable window into the largely neglected domain of neonatal SCIs [136].

## 9. Conclusions

SCI research has advanced considerably over the past four decades, yet many aspects of its pathophysiology and recovery mechanisms remain unresolved. The transient loss of reflexes during spinal shock, the propagation of injury potentials, and the early brain responses to SCI continue to represent critical blind spots that limit therapeutic progress. Supraspinal biomarkers may improve early diagnosis, prognostication, and monitoring of interventions, and there is emerging evidence for some of them, like NSE, S‐100β, and microRNAs, to carry therapeutic potential. At the same time, molecular and electrophysiological findings indicate that intrinsic spinal networks retain a greater capacity for functional recovery than traditionally assumed, as highlighted by pediatric cases and neonatal ex vivo models. Taken together, findings from our two ex vivo models of SCI in early development stages provide valuable insights into the dynamics of acute damage progression, from the immediate effects of trauma to the onset of secondary injury. Notably, two consecutive depolarization waves follow spinal cord trauma. The first injury potential is larger and faster, driven primarily by an ionic imbalance propagating through the extracellular space. The second wave is slower and longer‐lasting, likely triggered by a toxic over‐release of glutamate. Between 25 min and 24 h post‐lesion, locomotor circuits in the lumbar enlargement exhibit distinct level of dysfunction, particularly in integrating sensory afferent input. These data support a critical “double‐tap” mechanism underlying acute SCI progression: the initial mechanical trauma causes immediate structural damage and activates spared neural and glial elements, including early‐responding microglia. However, this first wave of cellular defense is rapidly overwhelmed by a second, delayed depolarizing insult, which exacerbates tissue damage and accelerates functional decline by aborting endogenous repair strategies. This two‐hit sequence, captured with unprecedented resolution in our ex vivo CNS models, offers a compelling framework to understand the rapid deterioration following SCI and highlights a narrow but actionable window for early therapeutic intervention. Importantly, the remaining spinal circuitry may still be exploited through targeted activation [133], provided that the gray matter downstream of the injury remains largely spared.

## Author Contributions

The first outline of the manuscript was written by Giuliano Taccola. The draft of the manuscript was written by Mariia E. Ermolaeva, Dimitry Sayenko and Giuliano Taccola, and illustrated by Mariia E. Ermolaeva and Atiyeh Mohammadshirazi. Atiyeh Mohammadshirazi commented on previous versions of the manuscript.

## Funding

Open access publishing facilitated by Scuola Internazionale Superiore di Studi Avanzati, as part of the Wiley ‐ CRUI‐CARE agreement.

## Disclosure

This manuscript has never been published as a pre‐print. All authors approved the final manuscript. All authors give their formal consent for the publication of the present manuscript.

## Consent

All authors give their formal consent to participate to the present manuscript.

## Conflicts of Interest

The authors declare no conflicts of interest.

## Data Availability

The impactor described in the study is currently being patented by SISSA, illustrated at the webpage https://www.valorisation.sissa.it/device-mechanically-stimulating-biological-material-and-its-procedure and available upon request.

## References

[1] Ahuja C. S. , Nori S. , and Tetreault L. , et al.Traumatic Spinal Cord Injury—Repair and Regeneration, Neurosurgery. (2017) 80, no. 3S, S9–S22, 10.1093/neuros/nyw080, 2-s2.0-85021635745. 28350947

[2] World Health Organization (WHO) , Spinal Cord Injury: A Global Perspective, 2024, World Health Organization Report.

[3] Guest J. , Datta N. , Jimsheleishvili G. , and Gater D. R.Jr., Pathophysiology, Classification and Comorbidities After Traumatic Spinal Cord Injury, Journal of Personalized Medicine. (2022) 12, no. 7, 10.3390/jpm12071126, 1126. 35887623 PMC9323191

[4] Bilchak J. N. , Caron G. , and Côté M.-P. , Exercise-Induced Plasticity in Signaling Pathways Involved in Motor Recovery After Spinal Cord Injury, International Journal of Molecular Sciences. (2021) 22, no. 9, 10.3390/ijms22094858, 4858. 34064332 PMC8124911

[5] Samejima S. , Henderson R. , Pradarelli J. , Mondello S. E. , and Moritz C. T. , Activity-Dependent Plasticity and Spinal Cord Stimulation for Motor Recovery Following Spinal Cord Injury, Experimental Neurology. (2022) 357, 10.1016/j.expneurol.2022.114178, 114178. 35878817

[6] Gad P. , Gerasimenko Y. , and Zdunowski S. , et al.Weight Bearing Over-ground Stepping in an Exoskeleton With Non-invasive Spinal Cord Neuromodulation After Motor Complete Paraplegia, Frontiers in Neuroscience. (2017) 11, 10.3389/fnins.2017.00333, 2-s2.0-85020749584, 333. 28642680 PMC5462970

[7] Lorach H. , Galvez A. , and Spagnolo V. , et al.Walking Naturally After Spinal Cord Injury Using a Brain–Spine Interface, Nature. (2023) 618, no. 7963, 126–133, 10.1038/s41586-023-06094-5. 37225984 PMC10232367

[8] Siddiqui A. M. , Khazaei M. , and Fehlings M. G. , Translating Mechanisms of Neuroprotection, Regeneration, and Repair to Treatment of Spinal Cord Injury, Progress in Brain Research. (2015) 218, 15–54, 10.1016/bs.pbr.2014.12.007, 2-s2.0-84955401993. 25890131

[9] Alizadeh A. , Dyck S. M. , and Karimi-Abdolrezaee S. , Traumatic Spinal Cord Injury: An Overview of Pathophysiology, Models and Acute Injury Mechanisms, Frontiers in Neurology. (2019) 10, 10.3389/fneur.2019.00282, 282. 30967837 PMC6439316

[10] MacFarlane M. P. and Glenn T. C. , Neurochemical Cascade of Concussion, Brain Injury. (2015) 29, no. 2, 139–153, 10.3109/02699052.2014.965208, 2-s2.0-84921292999. 25587743

[11] Tator C. H. and Fehlings M. G. , Review of the Secondary Injury Theory of Acute Spinal Cord Trauma With Emphasis on Vascular Mechanisms, Journal of Neurosurgery. (1991) 75, no. 1, 15–26, 10.3171/jns.1991.75.1.0015, 2-s2.0-0026326449. 2045903

[12] Carlson S. L. , Parrish M. E. , Springer J. E. , Doty K. , and Dossett L. , Acute Inflammatory Response in Spinal Cord Following Impact Injury, Experimental Neurology. (1998) 151, no. 1, 77–88, 10.1006/exnr.1998.6785, 2-s2.0-0032079920. 9582256

[13] Oyinbo C. , Secondary Injury Mechanisms in Traumatic Spinal Cord Injury: A Nugget of This Multiply Cascade, Acta Neurobiologiae Experimentalis. (2011) 71, no. 2, 281–299, 10.55782/ane-2011-1848. 21731081

[14] Leis A. A. , Kronenberg M. F. , Stetkarova I. , Paske W. C. , and Stokic D. S. , Spinal Motoneuron Excitability After Acute Spinal Cord Injury in Humans, Neurology. (1996) 47, no. 1, 231–237, 10.1212/WNL.47.1.231, 2-s2.0-0030054848. 8710084

[15] Hall M. , Second Memoir on Some Principles of the Pathology of the Nervous System, Medico-Chirurgical Transactions. (1840) 23, 121–167, 10.1177/095952874002300111. PMC2116903 20895701

[16] Sherrington C. S. , Address on the Spinal Animal, Medico-Chirurgical Transactions. (1899) 82, 449. 10.1177/095952879908200122 PMC2036689 20896941

[17] Simpson R. K.Jr., Robertson C. S. , and Goodman J. C. , The Role of Glycine in Spinal Shock, The Journal of Spinal Cord Medicine. (2016) 19, no. 4, 215–224, 10.1080/10790268.1996.11719437, 2-s2.0-0030252753. 9237788

[18] Ditunno J. F. , Little J. W. , Tessler A. , and Burns A. S. , Spinal Shock Revisited: A Four-Phase Model, Spinal Cord. (2004) 42, no. 7, 383–395, 10.1038/sj.sc.3101603, 2-s2.0-3242743582. 15037862

[19] McBride R. , Parker E. , and Garabed R. B. , et al.Developing a Predictive Model for Spinal Shock in Dogs With Spinal Cord Injury, Journal of Veterinary Internal Medicine. (2022) 36, no. 2, 663–671, 10.1111/jvim.16352. 35001437 PMC8965241

[20] Hall M. , Synopsis of the Diastaltic Nervous System, 1850, Mallett. 19301607

[21] Sherrington C. S. , Croonian Lecture—The Mammalian Spinal Cord as an Organ of Reflex Action, Proceedings of the Royal Society of London. (1897) 61, no. 369–377, 220–221, 10.1098/rspl.1897.0025.

[22] Sherrington C. S. , The Integrative Action of the Nervous System, 1906, Constable & Company Ltd..

[23] Jamjoom A. A. B. , Rhodes J. , Andrews P. J. D. , and Grant S. G. N. , The Synapse in Traumatic Brain Injury, Brain. (2021) 144, no. 1, 18–31, 10.1093/brain/awaa321. 33186462 PMC7880663

[24] Hoffe B. and Holahan M. R. , Hyperacute Excitotoxic Mechanisms and Synaptic Dysfunction Involved in Traumatic Brain Injury, Frontiers in Molecular Neuroscience. (2022) 15, 10.3389/fnmol.2022.831825, 831825. 35283730 PMC8907921

[25] Ping X. , Jiang K. , Lee S.-Y. , Cheng J.-X. , and Jin X. , PEG-PDLLA Micelle Treatment Improves Axonal Function of the Corpus Callosum Following Traumatic Brain Injury, Journal of Neurotrauma. (2014) 31, no. 13, 1172–1179, 10.1089/neu.2013.3147, 2-s2.0-84903827809. 24579802 PMC4082361

[26] Carron S. F. , Alwis D. S. , and Rajan R. , Traumatic Brain Injury and Neuronal Functionality Changes in Sensory Cortex, Frontiers in Systems Neuroscience. (2016) 10, 10.3389/fnsys.2016.00047, 2-s2.0-84989315057, 47. 27313514 PMC4889613

[27] Eidelberg E. , Sullivan J. , and Brigham A. , Immediate Consequences of Spinal Cord Injury: Possible Role of Potassium in Axonal Conduction Block, Surgical Neurology. (1975) 3, no. 6, 317–321. 1162585

[28] Nacimiento W. and Noth J. , What, if Anything, Is Spinal Shock?, Archives of Neurology. (1999) 56, no. 8, 1033–1035, 10.1001/archneur.56.8.1033, 2-s2.0-0032866809. 10448814

[29] Munteanu C. , Rotariu M. , and Turnea M. , et al.Main Cations and Cellular Biology of Traumatic Spinal Cord Injury, Cells. (2022) 11, no. 16, 10.3390/cells11162503, 2503. 36010579 PMC9406880

[30] Ko H. Y. , Spinal Shock: Understanding the Phenomenon and Reflex Recovery Patterns, A Practical Guide to Care of Spinal Cord Injuries: Clinical Questions and Answers, 2023, Springer Nature Singapore, 271–282.

[31] Fehlings M. G. and Perrin R. G. , The Role and Timing of Early Decompression for Cervical Spinal Cord Injury: Update With a Review of Recent Clinical Evidence, Injury. (2005) 36, no. 2, S13–S26, 10.1016/j.injury.2005.06.011, 2-s2.0-21244451106. 15993113

[32] Wang T. Y. , Park C. , and Zhang H. , et al.Management of Acute Traumatic Spinal Cord Injury: A Review of the Literature, Frontiers in Surgery. (2021) 8, 10.3389/fsurg.2021.698736, 698736. 34966774 PMC8710452

[33] Hu X. , Xu W. , and Ren Y. , et al.Spinal Cord Injury: Molecular Mechanisms and Therapeutic Interventions, Signal Transduction and Targeted Therapy. (2023) 8, no. 1, 10.1038/s41392-023-01477-6, 245. 37357239 PMC10291001

[34] Ziu E. , Weisbrod L. J. , and Mesfin F. B. , Spinal Shock, 2024, StatPearls Publishing. 28846241

[35] Gouveia D. , Cardoso A. , and Carvalho C. , et al.Influence of Spinal Shock on the Neurorehabilitation of ANNPE Dogs, Animals. (2022) 12, no. 12, 10.3390/ani12121557, 1557. 35739893 PMC9219513

[36] Ko H. Y. , Understanding Electrolyte Imbalances in Spinal Cord Injuries, A Practical Guide to Care of Spinal Cord Injuries: Clinical Questions and Answers, 2023, Springer Nature Singapore, 681–696.

[37] Grafstein B. , Mechanism of Spreading Cortical Depression, Journal of Neurophysiology. (1956) 19, no. 2, 154–171, 10.1152/jn.1956.19.2.154. 13295838

[38] Weilinger N. L. , Maslieieva V. , Bialecki J. , Sridharan S. S. , Tang P. L. , and Thompson R. J. , Ionotropic Receptors and Ion Channels in Ischemic Neuronal Death and Dysfunction, Acta Pharmacologica Sinica. (2013) 34, no. 1, 39–48, 10.1038/aps.2012.95, 2-s2.0-84872066398. 22864302 PMC4086487

[39] Grover H. , Qian Y. , Boada F. , and Lui Y. W. , Sodium Dysregulation in Traumatic Brain Injury, Cellular, Molecular, Physiological, and Behavioral Aspects of Traumatic Brain Injury, 2022, Academic Press, 257–266.

[40] Rodrigues T. , Piccirillo S. , and Magi S. , et al.Control of Ca2+ and Metabolic Homeostasis by the Na+/Ca2+ Exchangers (NCXs) in Health and Disease, Biochemical Pharmacology. (2022) 203, 10.1016/j.bcp.2022.115163, 115163. 35803319

[41] Boulenguez P. , Liabeuf S. , and Bos R. , et al.Down-Regulation of the Potassium-Chloride Cotransporter KCC2 Contributes to Spasticity after Spinal Cord Injury, Nature Medicine. (2010) 16, no. 3, 302–307, 10.1038/nm.2107, 2-s2.0-77949275173. 20190766

[42] Cramer S. W. , Baggott C. , and Cain J. , et al.The Role of Cation-Dependent Chloride Transporters in Neuropathic Pain Following Spinal Cord Injury, Molecular Pain. (2008) 4, 1744–8069, 10.1186/1744-8069-4-36, 2-s2.0-53549122751. PMC2561007 18799000

[43] Stil A. , Liabeuf S. , Jean-Xavier C. , Brocard C. , Viemari J.-C. , and Vinay L. , Developmental up-Regulation of the Potassium–Chloride Cotransporter Type 2 in the Rat Lumbar Spinal Cord, Neuroscience. (2009) 164, no. 2, 809–821, 10.1016/j.neuroscience.2009.08.035, 2-s2.0-70349745707. 19699273

[44] Vinay L. and Jean-Xavier C. , Plasticity of Spinal Cord Locomotor Networks and Contribution of Cation–Chloride Cotransporters, Brain Research Reviews. (2008) 57, no. 1, 103–110, 10.1016/j.brainresrev.2007.09.003, 2-s2.0-36749070905. 17949820

[45] Coull J. A. M. , Beggs S. , and Boudreau D. , et al.BDNF From Microglia Causes the Shift in Neuronal Anion Gradient Underlying Neuropathic Pain, Nature. (2005) 438, no. 7070, 1017–1021, 10.1038/nature04223, 2-s2.0-30744439794. 16355225

[46] Gwak Y. S. and Hulsebosch C. E. , Neuronal Hyperexcitability: A Substrate for Central Neuropathic Pain After Spinal Cord Injury, Current Pain and Headache Reports. (2011) 15, no. 3, 215–222, 10.1007/s11916-011-0186-2, 2-s2.0-79955919643. 21387163

[47] Chen S. , Siedhoff H. R. , and Zhang H. , et al.Low-Intensity Blast Induces Acute Glutamatergic Hyperexcitability in Mouse Hippocampus Leading to Long-Term Learning Deficits and Altered Expression of Proteins Involved in Synaptic Plasticity and Serine Protease Inhibitors, Neurobiology of Disease. (2022) 165, 10.1016/j.nbd.2022.105634, 105634. 35077822 PMC12928849

[48] Wolf J. A. , Stys P. K. , Lusardi T. , Meaney D. , and Smith D. H. , Traumatic Axonal Injury Induces Calcium Influx Modulated by Tetrodotoxin-Sensitive Sodium Channels, Journal of Neuroscience. (2001) 21, no. 6, 1923–1930, 10.1523/JNEUROSCI.21-06-01923.2001. 11245677 PMC6762603

[49] Sukumaran P. , Nascimento Da Conceicao V. , and Sun Y. , et al.Calcium Signaling Regulates Autophagy and Apoptosis, Cells. (2021) 10, no. 8, 10.3390/cells10082125, 2125. 34440894 PMC8394685

[50] Dreier J. P. , The Role of Spreading Depression, Spreading Depolarization and Spreading Ischemia in Neurological Disease, Nature Medicine. (2011) 17, no. 4, 439–447, 10.1038/nm.2333, 2-s2.0-79953757268. 21475241

[51] Gao B. X. and Ziskind-Conhaim L. , Development of Glycine-and GABA-Gated Currents in Rat Spinal Motoneurons, Journal of Neurophysiology. (1995) 74, no. 1, 113–121, 10.1152/jn.1995.74.1.113, 2-s2.0-0029085344. 7472315

[52] Mazzone G. L. , Mohammadshirazi A. , Aquino J. B. , Nistri A. , and Taccola G. , GABAergic Mechanisms Can Redress the Tilted Balance Between Excitation and Inhibition in Damaged Spinal Networks, Molecular Neurobiology. (2021) 58, no. 8, 3769–3786, 10.1007/s12035-021-02370-5. 33826070 PMC8279998

[53] Leao A. A. P. , Spreading Depression of Activity in the Cerebral Cortex, Journal of Neurophysiology. (1944) 7, no. 6, 359–390, 10.1152/jn.1944.7.6.359. 20268874

[54] Carmichael S. T. , The 3 Rs of Stroke Biology: Radial, Relayed, and Regenerative, Neurotherapeutics. (2016) 13, no. 2, 348–359, 10.1007/s13311-015-0408-0, 2-s2.0-84948127794. 26602550 PMC4824028

[55] Lauritzen M. , Dreier J. P. , Fabricius M. , Hartings J. A. , Graf R. , and Strong A. J. , Clinical Relevance of Cortical Spreading Depression in Neurological Disorders: Migraine, Malignant Stroke, Subarachnoid and Intracranial Hemorrhage, and Traumatic Brain Injury, Journal of Cerebral Blood Flow & Metabolism. (2011) 31, no. 1, 17–35, 10.1038/jcbfm.2010.191, 2-s2.0-78650895470. 21045864 PMC3049472

[56] Somjen G. G. , Mechanisms of Spreading Depression and Hypoxic Spreading Depression-Like Depolarization, Physiological Reviews. (2001) 81, no. 3, 1065–1096, 10.1152/physrev.2001.81.3.1065, 2-s2.0-0034953204. 11427692

[57] Kraio R. P. and Nicholson C. , Extracellular Ionic Variations During Spreading Depression, Neuroscience. (1978) 3, no. 11, 1045–1059, 10.1016/0306-4522(78)90122-7, 2-s2.0-0018091909. 745780

[58] Gerasimova E. , Burkhanova G. , and Chernova K. , et al.Hyperhomocysteinemia Increases Susceptibility to Cortical Spreading Depression Associated With Photophobia, Mechanical Allodynia, and Anxiety in Rats, Behavioural Brain Research. (2021) 409, 10.1016/j.bbr.2021.113324, 113324. 33915239

[59] Shatillo A. , Salo R. A. , Giniatullin R. , and Gröhn O. H. , Involvement of NMDA Receptor Subtypes in Cortical Spreading Depression in Rats Assessed by fMRI, Neuropharmacology. (2015) 93, 164–170, 10.1016/j.neuropharm.2015.01.028, 2-s2.0-84923622323. 25688928

[60] Hermann D. M. , Mies G. , and Hossmann K.-A. , Biochemical Changes and Gene Expression Following Traumatic Brain Injury: Role of SpReading Depression, Restorative Neurology and Neuroscience. (1999) 14, no. 2-3, 103–108, 10.3233/RNN-1999-00082. 22387505

[61] Harreveld A. V. , Compounds in Brain Extracts Causing Spreading Depression of Cerebral Cortical Activity and Contraction of Crustacean Muscle, Journal of Neurochemistry. (1959) 3, no. 4, 300–315, 10.1111/j.1471-4159.1959.tb12636.x, 2-s2.0-52549102652. 13642064

[62] Wendt S. , Wogram E. , Korvers L. , and Kettenmann H. , Experimental Cortical Spreading Depression Induces NMDA Receptor Dependent Potassium Currents in Microglia, Journal of Neuroscience. (2016) 36, no. 23, 6165–6174, 10.1523/JNEUROSCI.4498-15.2016, 2-s2.0-84973322883. 27277795 PMC6604883

[63] Lindquist B. E. , Spreading Depolarizations Pose Critical Energy Challenges in Acute Brain Injury, Journal of Neurochemistry. (2024) 168, no. 5, 868–887, 10.1111/jnc.15966. 37787065 PMC10987398

[64] Magi S. , Piccirillo S. , Amoroso S. , and Lariccia V. , Excitatory Amino Acid Transporters (EAATs): Glutamate Transport and Beyond, International Journal of Molecular Sciences. (2019) 20, no. 22, 10.3390/ijms20225674, 5674. 31766111 PMC6888595

[65] Kettenmann H. , Banati R. , and Walz W. , Electrophysiological Behavior of Microglia, Glia. (1993) 7, no. 1, 93–101, 10.1002/glia.440070115, 2-s2.0-0027343512. 7678582

[66] Kearns K. N. , Liu L. , and Soldozy S. , et al.Microglia Modulate Cortical Spreading Depolarizations After Ischemic Stroke: A Narrative Review, Neurocritical Care. (2022) 37, no. S1, 133–138, 10.1007/s12028-022-01469-4. 35288861 PMC9259539

[67] Ayata C. and Lauritzen M. , Spreading Depression, Spreading Depolarizations, and the Cerebral Vasculature, Physiological Reviews. (2015) 95, no. 3, 953–993, 10.1152/physrev.00027.2014, 2-s2.0-84936854483. 26133935 PMC4491545

[68] Streit D. S. , Ferreira Filho C. R. , and Martins-Ferreira H. , Spreading Depression in Isolated Spinal Cord, Journal of Neurophysiology. (1995) 74, no. 2, 888–890, 10.1152/jn.1995.74.2.888, 2-s2.0-0029162750. 7472391

[69] Gorji A. , Zahn P. K. , Pogatzki E. M. , and Speckmann E.-J. , Spinal and Cortical Spreading Depression Enhance Spinal Cord Activity, Neurobiology of Disease. (2004) 15, no. 1, 70–79, 10.1016/j.nbd.2003.09.014, 2-s2.0-1642578922. 14751772

[70] Crawley A. P. , Jurkiewicz M. T. , and Yim A. , et al.Absence of Localized Grey Matter Volume Changes in the Motor Cortex Following Spinal Cord Injury, Brain Research. (2004) 1028, no. 1, 19–25, 10.1016/j.brainres.2004.08.060, 2-s2.0-7044286033. 15518637

[71] Feringa E. R. , Lee G. W. , and Vahlsing H. L. , Cell Death in Clarke’s Column After Spinal Cord Transection, Journal of Neuropathology and Experimental Neurology. (1985) 44, no. 2, 156–164, 10.1097/00005072-198503000-00004, 2-s2.0-0021988509. 3973636

[72] Hains B. C. , Black J. A. , and Waxman S. G. , Primary Cortical Motor Neurons Undergo Apoptosis After Axotomizing Spinal Cord Injury, Journal of Comparative Neurology. (2003) 462, no. 3, 328–341, 10.1002/cne.10733, 2-s2.0-0038649642. 12794736

[73] Aguilar J. , Humanes-Valera D. , and Alonso-Calviño E. , et al.Spinal Cord Injury Immediately Changes the State of the Brain, Journal of Neuroscience. (2010) 30, no. 22, 7528–7537, 10.1523/JNEUROSCI.0379-10.2010, 2-s2.0-77953225603. 20519527 PMC3842476

[74] Wu J. , Zhao Z. , and Sabirzhanov B. , et al.Spinal Cord Injury Causes Brain Inflammation Associated With Cognitive and Affective Changes: Role of Cell Cycle Pathways, Journal of Neuroscience. (2014) 34, no. 33, 10989–11006, 10.1523/JNEUROSCI.5110-13.2014, 2-s2.0-84905825629. 25122899 PMC4131014

[75] Bonizzato M. , Pidpruzhnykova G. , and DiGiovanna J. , et al.Brain-Controlled Modulation of Spinal Circuits Improves Recovery From Spinal Cord Injury, Nature Communications. (2018) 9, no. 1, 10.1038/s41467-018-05282-6, 2-s2.0-85050959160, 3015. PMC6070513 30068906

[76] Bonizzato M. and Martinez M. , An Intracortical Neuroprosthesis Immediately Alleviates Walking Deficits and Improves Recovery of Leg Control after Spinal Cord Injury, Science Translational Medicine. (2021) 13, no. 586, 10.1126/scitranslmed.abb4422, eabb4422. 33762436

[77] Lemieux M. , Karimi N. , and Bretzner F. , Functional Plasticity of Glutamatergic Neurons of Medullary Reticular Nuclei After Spinal Cord Injury in Mice, Nature Communications. (2024) 15, no. 1, 10.1038/s41467-024-45300-4, 1542. PMC10879492 38378819

[78] Hill C. E. , A View From the Ending: Axonal Dieback and Regeneration Following SCI, Neuroscience Letters. (2017) 652, 11–24, 10.1016/j.neulet.2016.11.002, 2-s2.0-85008502058. 27825985

[79] Hsu L.-J. , Bertho M. , and Kiehn O. , Deconstructing the Modular Organization and Real-Time Dynamics of Mammalian Spinal Locomotor Networks, Nature Communications. (2023) 14, no. 1, 10.1038/s41467-023-36587-w, 873. PMC9935527 36797254

[80] Bennett D. J. , Gorassini M. , Fouad K. , Sanelli L. , Han Y. , and Cheng J. , Spasticity in Rats With Sacral Spinal Cord Injury, Journal of Neurotrauma. (1999) 16, no. 1, 69–84, 10.1089/neu.1999.16.69, 2-s2.0-0032941892. 9989467

[81] Carlton S. M. , Du J. , and Tan H. Y. , et al.Peripheral and Central Sensitization in Remote Spinal Cord Regions Contribute to Central Neuropathic Pain After Spinal Cord Injury, Pain. (2009) 147, no. 1, 265–276, 10.1016/j.pain.2009.09.030, 2-s2.0-70449622719. 19853381 PMC2787843

[82] Latremoliere A. and Woolf C. J. , Central Sensitization: A Generator of Pain Hypersensitivity by Central Neural Plasticity, The Journal of Pain. (2009) 10, no. 9, 895–926, 10.1016/j.jpain.2009.06.012, 2-s2.0-68949189686. 19712899 PMC2750819

[83] Xiong W. , Ping X. , and Ripsch M. S. , et al.Enhancing Excitatory Activity of Somatosensory Cortex Alleviates Neuropathic Pain Through Regulating Homeostatic Plasticity, Scientific Reports. (2017) 7, no. 1, 10.1038/s41598-017-12972-6, 2-s2.0-85030748835, 12743. 28986567 PMC5630599

[84] Calancie B. , Broton J. G. , Klose K. J. , Traad M. , Difini J. , and Ayyar D. R. , Evidence That Alterations in Presynaptic Inhibition Contribute to Segmental Hypo-and Hyperexcitability After Spinal Cord Injury in Man, Electroencephalography and Clinical Neurophysiology/Evoked Potentials Section. (1993) 89, no. 3, 177–186, 10.1016/0168-5597(93)90131-8, 2-s2.0-0027157907. 7686850

[85] Yague J. G. , Foffani G. , and Aguilar J. , Cortical Hyperexcitability in Response to Preserved Spinothalamic Inputs Immediately After Spinal Cord Hemisection, Experimental Neurology. (2011) 227, no. 2, 252–263, 10.1016/j.expneurol.2010.11.011, 2-s2.0-78651441820. 21093438

[86] Zaforas M. , Rosa J. M. , and Alonso-Calviño E. , et al.Cortical Layer-Specific Modulation of Neuronal Activity After Sensory Deprivation due to Spinal Cord Injury, Journal of Physiology. (2021) 599, no. 20, 4643–4669, 10.1113/JP281901. 34418097 PMC9292026

[87] Pouw M. H. , Hosman A. J. F. , Van Middendorp J. J. , Verbeek M. M. , Vos P. E. , and van de Meent H. , Biomarkers in Spinal Cord Injury, Spinal Cord. (2009) 47, no. 7, 519–525, 10.1038/sc.2008.176, 2-s2.0-67651177464. 19153591

[88] Pouw M. H. , Kwon B. K. , and Verbeek M. M. , et al.Structural Biomarkers in the Cerebrospinal Fluid Within 24 h After a Traumatic Spinal Cord Injury: A Descriptive Analysis of 16 Subjects, Spinal Cord. (2014) 52, no. 6, 428–433, 10.1038/sc.2014.26, 2-s2.0-84902268189. 24710150

[89] Kwon B. K. , Bloom O. , and Wanner I. B. , et al.Neurochemical Biomarkers in Spinal Cord Injury, Spinal Cord. (2019) 57, no. 10, 819–831, 10.1038/s41393-019-0319-8, 2-s2.0-85068833788. 31273298

[90] Nagy G. , Dzsinich C. , and Selmeci L. , et al.Biochemical Alterations in Cerebrospinal Fluid During Thoracoabdominal Aortic Cross-Clamping in Dogs, Annals of Vascular Surgery. (2002) 16, no. 4, 436–441, 10.1007/s10016-001-0037-4, 2-s2.0-0036628775. 12089629

[91] Cao F. , Yang X. F. , and Liu W. G. , et al.Elevation of Neuron-Specific Enolase and S-100β Protein Level in Experimental Acute Spinal Cord Injury, Journal of Clinical Neuroscience. (2008) 15, no. 5, 541–544, 10.1016/j.jocn.2007.05.014, 2-s2.0-41549164237. 18343116

[92] Nasser M. , Bejjani F. , and Raad M. , et al.Traumatic Brain Injury and Blood-Brain Barrier Cross-Talk, CNS & Neurological Disorders - Drug Targets. (2016) 15, no. 9, 1030–1044, 10.2174/1871527315666160815093525. 27528468

[93] Mazzone G. L. and Nistri A. , S100β as an Early Biomarker of Excitotoxic Damage in Spinal Cord Organotypic Cultures, Journal of Neurochemistry. (2014) 130, no. 4, 598–604, 10.1111/jnc.12748, 2-s2.0-84905730566. 24766228

[94] Mio L. , Pistorio G. , Mohammadshirazi A. , Taccola G. , and Falcone C. , Immediate Cortical Glial Alterations Following Spinal Cord Injury: Evidence From a Novel In Vitro Model, Experimental Physiology. (2025) 6, 1–6, 10.1113/EP092809. PMC13140715 41054235

[95] Rodrigues L. F. , Moura-Neto V. , and de Sampaio E Spohr T. C. L. , Biomarkers in Spinal Cord Injury: From Prognosis to Treatment, Molecular Neurobiology. (2018) 55, no. 8, 6436–6448, 10.1007/s12035-017-0858-y, 2-s2.0-85040051863. 29307082

[96] Di Pietro L. , Baranzini M. , and Berardinelli M. G. , et al.Potential Therapeutic Targets for ALS: MIR206, MIR208b and MIR499 Are Modulated During Disease Progression in the Skeletal Muscle of Patients, Scientific Reports. (2017) 7, no. 1, 10.1038/s41598-017-10161-z, 2-s2.0-85028311984, 9538. 28842714 PMC5573384

[97] Pinchi E. , Luigi C. , and Paola S. , et al.MicroRNAs: The New Challenge for Traumatic Brain Injury Diagnosis, Current Neuropharmacology. (2020) 18, no. 4, 319–331, 10.2174/1570159X17666191113100808. 31729300 PMC7327940

[98] Benedetti B. , Bieler L. , and Erhardt-Kreutzer C. , et al.Depolarization and Hyperexcitability of Cortical Motor Neurons After Spinal Cord Injury Associates With Reduced HCN Channel Activity, International Journal of Molecular Sciences. (2023) 24, no. 5, 10.3390/ijms24054715, 4715. 36902146 PMC10003573

[99] Najem D. , Rennie K. , and Ribecco-Lutkiewicz M. , et al.Traumatic Brain Injury: Classification, Models, and Markers, Biochemistry and Cell Biology. (2018) 96, no. 4, 391–406, 10.1139/bcb-2016-0160, 2-s2.0-85051328453. 29370536

[100] Kirshblum S. , Snider B. , Eren F. , and Guest J. , Characterizing Natural Recovery After Traumatic Spinal Cord Injury, Journal of Neurotrauma. (2021) 38, no. 9, 1267–1284, 10.1089/neu.2020.7473. 33339474 PMC8080912

[101] Geisler F. H. , Coleman W. P. , Grieco G. , and Poonian D. , Measurements and Recovery Patterns in a Multicenter Study of Acute Spinal Cord Injury, Spine. (2001) 26, no. S1, S68–S86, 10.1097/00007632-200112151-00014. 11805613

[102] Van den Brand R. , Heutschi J. , and Barraud Q. , et al.Restoring Voluntary Control of Locomotion After Paralyzing Spinal Cord Injury, Science. (2012) 336, no. 6085, 1182–1185, 10.1126/science.1217416, 2-s2.0-84861696705. 22654062

[103] Friedli L. , Rosenzweig E. S. , and Barraud Q. , et al.Pronounced Species Divergence in Corticospinal Tract Reorganization and Functional Recovery After Lateralized Spinal Cord Injury Favors Primates, Science Translational Medicine. (2015) 7, no. 302, 10.1126/scitranslmed.aac5811, 2-s2.0-84940416986, 302ra134. PMC5669362 26311729

[104] Liu Y. , Wang X. , and Li W. , et al.A Sensitized IGF1 Treatment Restores Corticospinal Axon-Dependent Functions, Neuron. (2017) 95, no. 4, 817–833.e4, 10.1016/j.neuron.2017.07.037, 2-s2.0-85027457606. 28817801 PMC5582621

[105] Cho N. , Squair J. W. , and Aureli V. , et al.Hypothalamic Deep Brain Stimulation Augments Walking After Spinal Cord Injury, Nature Medicine. (2024) 30, 3676–3686. 10.1038/s41591-024-03306-x 39623087

[106] Siegel C. S. , Fink K. L. , Strittmatter S. M. , and Cafferty W. B. J. , Plasticity of Intact Rubral Projections Mediates Spontaneous Recovery of Function After Corticospinal Tract Injury, Journal of Neuroscience. (2015) 35, no. 4, 1443–1457, 10.1523/JNEUROSCI.3713-14.2015, 2-s2.0-84921908837. 25632122 PMC4308593

[107] Bracken M. B. , Shepard M. J. , and Holford T. R. , et al.Methylprednisolone or Tirilazad Mesylate Administration After Acute Spinal Cord Injury: 1-Year Follow up: Results of the Third National Acute Spinal Cord Injury Randomized Controlled Trial, Journal of Neurosurgery. (1998) 89, no. 5, 699–706, 10.3171/jns.1998.89.5.0699, 2-s2.0-17144468967. 9817404

[108] Li L. S. , Yu H. , and Raynald R. , et al.Anatomical Mechanism of Spontaneous Recovery in Regions Caudal to Thoracic Spinal Cord Injury Lesions in Rats, PeerJ. (2017) 5. 10.7717/peerj.2865 PMC5228130 28097067

[109] Fawcett J. W. , Curt A. , and Steeves J. D. , et al.Guidelines for the Conduct of Clinical Trials for Spinal Cord Injury as Developed by the ICCP Panel: Spontaneous Recovery After Spinal Cord Injury and Statistical Power Needed for Therapeutic Clinical Trials, Spinal Cord. (2007) 45, no. 3, 190–205, 10.1038/sj.sc.3102007, 2-s2.0-33847391178. 17179973

[110] de la Barrera S. S. , Barca-Buyo A. , Montoto-Marqués A. , Ferreiro-Velasco M. E. , Cidoncha-Dans M. , and Rodriguez-Sotillo A. , Spinal Cord Infarction: Prognosis and Recovery in a Series of 36 Patients, Spinal Cord. (2001) 39, no. 10, 520–525, 10.1038/sj.sc.3101201, 2-s2.0-0034775471. 11641795

[111] Skinnider M. A. , Gautier M. , and Teo A. Y. Y. , et al.Single-Cell and Spatial Atlases of Spinal Cord Injury in the Tabulae Paralytica, Nature. (2024) 631, no. 8019, 150–163, 10.1038/s41586-024-07504-y. 38898272

[112] Oudega M. , Vargas C. G. , Weber A. B. , Kleitman N. , and Bunge M. B. , Long-Term Effects of Methylprednisolone Following Transection of Adult Rat Spinal Cord, The European Journal of Neuroscience. (1999) 11, no. 7, 2453–2464, 10.1046/j.1460-9568.1999.00666.x, 2-s2.0-0032971226. 10383635

[113] Sasaki M. , Hains B. C. , Lankford K. L. , Waxman S. G. , and Kocsis J. D. , Protection of Corticospinal Tract Neurons After Dorsal Spinal Cord Transection and Engraftment of Olfactory Ensheathing Cells, Glia. (2006) 53, no. 4, 352–359, 10.1002/glia.20285, 2-s2.0-32544460761. 16288464 PMC2605395

[114] Ghosh A. , Haiss F. , and Sydekum E. , et al.Rewiring of Hindlimb Corticospinal Neurons After Spinal Cord Injury, Nature Neuroscience. (2010) 13, no. 1, 97–104, 10.1038/nn.2448, 2-s2.0-73949107473. 20010824

[115] Ghosh S. and Hui S. P. , Axonal Regeneration in Zebrafish Spinal Cord, Regeneration. (2018) 5, no. 1, 43–60, 10.1002/reg2.99. 29721326 PMC5911453

[116] Tran A. P. , Warren P. M. , and Silver J. , The Biology of Regeneration Failure and Success After Spinal Cord Injury, Physiological Reviews. (2018) 98, no. 2, 881–917, 10.1152/physrev.00017.2017, 2-s2.0-85043598011. 29513146 PMC5966716

[117] Hilton B. J. , Anenberg E. , Harrison T. C. , Boyd J. D. , Murphy T. H. , and Tetzlaff W. , Re-Establishment of Cortical Motor Output Maps and Spontaneous Functional Recovery via Spared Dorsolaterally Projecting Corticospinal Neurons After Dorsal Column Spinal Cord Injury in Adult Mice, The Journal of Neuroscience. (2016) 36, no. 14, 4080–4092, 10.1523/JNEUROSCI.3386-15.2016, 2-s2.0-84964052741. 27053214 PMC6705513

[118] Weidner N. , Ner A. , Salimi N. , and Tuszynski M. H. , Spontaneous Corticospinal Axonal Plasticity and Functional Recovery After Adult Central Nervous System Injury, Proceedings of the National Academy of Sciences. (2001) 98, no. 6, 3513–3518, 10.1073/pnas.051626798, 2-s2.0-0035853114. PMC30684 11248109

[119] Filli L. and Schwab M. E. , Structural and Functional Reorganization of Propriospinal Connections Promotes Functional Recovery After Spinal Cord Injury, Neural Regeneration Research. (2015) 10, no. 4, 509–513, 10.4103/1673-5374.155425, 2-s2.0-84928812654. 26170799 PMC4424731

[120] Cazalets J. R. , Sqalli-Houssaini Y. , and Clarac F. , Activation of the Central Pattern Generators for Locomotion by Serotonin and Excitatory Amino Acids in Neonatal Rat, Journal of Physiology. (1992) 455, no. 1, 187–204, 10.1113/jphysiol.1992.sp019296, 2-s2.0-0026737329. 1362441 PMC1175639

[121] Pearson K. G. and Rossignol S. , Fictive Motor Patterns in Chronic Spinal Cats, Journal of Neurophysiology. (1991) 66, no. 6, 1874–1887, 10.1152/jn.1991.66.6.1874, 2-s2.0-0026353375. 1812222

[122] Schmidt B. J. and Jordan L. M. , The Role of Serotonin in Reflex Modulation and Locomotor Rhythm Production in the Mammalian Spinal Cord, Brain Research Bulletin. (2000) 53, no. 5, 689–710. 11165804 10.1016/s0361-9230(00)00402-0

[123] Kiehn O. and Kjaerulff O. , Spatiotemporal Characteristics of 5-HT and Dopamine-Induced Rhythmic Hindlimb Activity in the In Vitro Neonatal Rat, Journal of Neurophysiology. (1996) 75, no. 4, 1472–1482, 10.1152/jn.1996.75.4.1472, 2-s2.0-0029862950. 8727391

[124] Nakanishi S. T. and Whelan P. J. , A Decerebrate In Vivo Mouse Model for Examining the Sensorimotor Control of Locomotion, Journal of Neurophysiology. (2011) 107, 500–515. 21994265 10.1152/jn.00699.2011

[125] Murray K. C. , Nakae A. , and Stephens M. J. , et al.Recovery of Motoneuron and Locomotor Function After Spinal Cord Injury Depends on Constitutive Activity in 5-HT2C Receptors, Nature Medicine. (2010) 16, no. 6, 694–700, 10.1038/nm.2160, 2-s2.0-77953229364. PMC3107820 20512126

[126] Rossignol S. and Frigon A. , Recovery of Locomotion After Spinal Cord Injury: Some Facts and Mechanisms, Annual Review of Neuroscience. (2011) 34, no. 1, 413–440, 10.1146/annurev-neuro-061010-113746, 2-s2.0-79959865503. 21469957

[127] Courtine G. , Gerasimenko Y. , and Van Den Brand R. , et al.Transformation of Nonfunctional Spinal Circuits Into Functional States After the Loss of Brain Input, Nature Neuroscience. (2009) 12, no. 10, 1333–1342, 10.1038/nn.2401, 2-s2.0-70349524957. 19767747 PMC2828944

[128] Raineteau O. and Schwab M. E. , Plasticity of Motor Systems After Incomplete Spinal Cord Injury, Nature Reviews Neuroscience. (2001) 2, no. 4, 263–273, 10.1038/35067570, 2-s2.0-0035319602. 11283749

[129] Lynskey J. V. , Belanger A. , and Jung R. , Activity-Dependent Plasticity in Spinal Cord Injury, Journal of Rehabilitation Research and Development. (2008) 45, no. 2, 229–240, 10.1682/JRRD.2007.03.0047, 2-s2.0-57649203308. 18566941 PMC2562625

[130] Hassannejad Z. , Shakouri-Motlagh A. , and Mokhatab M. , et al.Oligodendrogliogenesis and Axon Remyelination After Traumatic Spinal Cord Injuries in Animal Studies: A Systematic Review, Neuroscience. (2019) 402, 37–50, 10.1016/j.neuroscience.2019.01.019, 2-s2.0-85061035614. 30685542

[131] Russ D. E. , Cross R. B. P. , and Li L. , et al.A Harmonized Atlas of Mouse Spinal Cord Cell Types and Their Spatial Organization, Nature Communications. (2021) 12, no. 1, 10.1038/s41467-021-25125-1, 5722. PMC8481483 34588430

[132] Matson K. J. , Russ D. E. , and Kathe C. , et al.Single Cell Atlas of Spinal Cord Injury in Mice Reveals a pro-Regenerative Signature in Spinocerebellar Neurons, Nature Communications. (2022) 13, no. 1, 10.1038/s41467-022-33184-1, 5628. PMC9513082 36163250

[133] Taccola G. , Sayenko D. , Gad P. , Gerasimenko Y. , and Edgerton V. R. , And Yet It Moves: Recovery of Volitional Control After Spinal Cord Injury, Progress in Neurobiology. (2018) 160, 64–81, 10.1016/j.pneurobio.2017.10.004, 2-s2.0-85033560970. 29102670 PMC5773077

[134] Taccola G. , Kissane R. , and Culaclii S. , et al.Dynamic Electrical Stimulation Enhances the Recruitment of Spinal Interneurons by Corticospinal Input, Experimental Neurology. (2024) 371, 10.1016/j.expneurol.2023.114589, 114589. 37907125

[135] Taccola G. , Steele A. G. , and Apicella R. , et al.Interactions Between Descending and Spinal Circuits on Motor Output, Experimental Neurology. (2025) 392, 10.1016/j.expneurol.2025.115347, 115347. 40505828 PMC12225539

[136] Carreon L. Y. , Glassman S. D. , and Campbell M. J. , Pediatric Spine Fractures: A Review of 137 Hospital Admissions, Journal of Spinal Disorders & Techniques. (2004) 17, no. 6, 477–482, 10.1097/01.bsd.0000132290.50455.99, 2-s2.0-16644380286. 15570118

[137] Eleraky M. A. , Theodore N. , Adams M. , Rekate H. L. , and Sonntag V. K. , Pediatric Cervical Spine Injuries: Report of 102 Cases and Review of the Literature, Journal of Neurosurgery. (2000) 92, no. 1 Suppl, 12–17, 10.3171/spi.2000.92.1.0012. 10616052

[138] Wang M. Y. , Hoh D. J. , Leary S. P. , Griffith P. , and McComb J. G. , High Rates of Neurological Improvement Following Severe Traumatic Pediatric Spinal Cord Injury, Spine. (2004) 29, no. 13, 1493–1497, 10.1097/01.BRS.0000129026.03194.0F, 2-s2.0-3042770778. 15223946

[139] Clarke E. C. , Cheng S. , and Bilston L. E. , The Mechanical Properties of Neonatal Rat Spinal Cord In Vitro, and Comparisons With Adult, Journal of Biomechanics. (2009) 42, no. 10, 1397–1402, 10.1016/j.jbiomech.2009.04.008, 2-s2.0-67649464254. 19442976

[140] Parent S. , Dimar J. , Dekutoski M. , and Roy-Beaudry M. , Unique Features of Pediatric Spinal Cord Injury, Spine. (2010) 35, no. S1, S202–S208, 10.1097/BRS.0b013e3181f35acb, 2-s2.0-77958021954. 20881463

[141] Huisman T. A. G. M. , Wagner M. W. , Bosemani T. , Tekes A. , and Poretti A. , Pediatric Spinal Trauma, Journal of Neuroimaging. (2015) 25, no. 3, 337–353, 10.1111/jon.12201, 2-s2.0-84929658129. 25512255

[142] Guttmann L. , Spinal Shock, Handbook of Clinical Neurology, 1976, 26, Elsevier, 243–262.

[143] Nicholls J. and Saunders N. , Regeneration of Immature Mammalian Spinal Cord After Injury, Trends in Neurosciences. (1996) 19, no. 6, 229–234, 10.1016/0166-2236(96)10021-7, 2-s2.0-0030011608. 8761958

[144] Jaerve A. , Schiwy N. , Schmitz C. , and Mueller H. W. , Differential Effect of Aging on Axon Sprouting and Regenerative Growth in Spinal Cord Injury, Experimental Neurology. (2011) 231, no. 2, 284–294, 10.1016/j.expneurol.2011.07.002, 2-s2.0-80052532414. 21806987

[145] Woodward S. K. A. , Treherne J. M. , Knott G. W. , Fernandez J. , Varga Z. M. , and Nicholls J. G. , Development of Connections by Axons Growing Through Injured Spinal Cord of Neonatal Opossum in Culture, Journal of Experimental Biology. (1993) 176, no. 1, 77–88, 10.1242/jeb.176.1.77. 8478604

[146] Bregman B. S. , Kunkel-Bagden E. , Schnell L. , Dai H. N. , Gao D. , and Schwab M. E. , Recovery From Spinal Cord Injury Mediated by Antibodies to Neurite Growth Inhibitors, Nature. (1995) 378, no. 6556, 498–501, 10.1038/378498a0, 2-s2.0-0028867947. 7477407

[147] Capuz A. , Karnoub M.-A. , and Osien S. , et al.The Antibody Dependant Neurite Outgrowth Modulation Response Involvement in Spinal Cord Injury, Frontiers in Immunology. (2022) 13, 10.3389/fimmu.2022.882830, 882830. 35784350 PMC9245426

[148] Wakabayashi Y. , Komori H. , and Kawa-Uchi T. , et al.Functional Recovery and Regeneration of Descending Tracts in Rats After Spinal Cord Transection in Infancy, Spine. (2001) 26, no. 11, 1215–1222, 10.1097/00007632-200106010-00009, 2-s2.0-0035370046. 11389386

[149] Bregman B. S. and Goldberger M. E. , Anatomical Plasticity and Sparing of Function After Spinal Cord Damage in Neonatal Cats, Science. (1982) 217, no. 4559, 553–555, 10.1126/science.7089581, 2-s2.0-0020066612. 7089581

[150] Kitade K. , Kobayakawa K. , and Saiwai H. , et al.Reduced Neuroinflammation Via Astrocytes and Neutrophils Promotes Regeneration After Spinal Cord Injury in Neonatal Mice, Journal of Neurotrauma. (2023) 40, no. 23-24, 2566–2579, 10.1089/neu.2023.0044. 37503626

[151] Jiang Q. , Xue S. , and Pan X. , et al.Differential Changes in the Microglial Transcriptome Between Neonatal and Adult Mice After Spinal Cord Injury, Scientific Reports. (2025) 15, no. 1, 10.1038/s41598-025-98429-7, 13708. 40258965 PMC12012053

[152] Stewart A. N. , Glaser E. P. , Bailey W. M. , and Gensel J. C. , Immunoglobulin G Is Increased in the Injured Spinal Cord in a Sex- and Age-Dependent Manner, Journal of Neurotrauma. (2022) 39, no. 15-16, 1090–1098, 10.1089/neu.2022.0011. 35373588 PMC9347383

[153] Ahuja C. S. , Wilson J. R. , and Nori S. , et al.Traumatic Spinal Cord Injury, Nature Reviews Disease Primers. (2017) 3, no. 1, 1–21, 10.1038/nrdp.2017.18, 2-s2.0-85018891841. 28447605

[154] Cunha N. S. C. , Malvea A. , Sadat S. , Ibrahim G. M. , and Fehlings M. G. , Pediatric Spinal Cord Injury: A Review, Children. (2023) 10, no. 9, 10.3390/children10091456, 1456. 37761417 PMC10530251

[155] Zheng J. and Nan G. , Advance in Pediatric Spinal Cord Injury, Pediatric Discovery. (2024) 2, no. 1, 10.1002/pdi3.55, e55. 40626248 PMC12118219

[156] Allen A. R. , Surgery of Experimental Lesion of Spinal Cord Equivalent to Crush Injury of Fracture Dislocation of Spinal Column: A Preliminary Report, Journal of the American Medical Association. (1911) LVII, no. 11, 878–880, 10.1001/jama.1911.04260090100008, 2-s2.0-84943442523.

[157] Wrathall J. R. , Pettegrew R. K. , and Harvey F. , Spinal Cord Contusion in the Rat: Production of Graded, Reproducible, Injury Groups, Experimental Neurology. (1985) 88, no. 1, 108–122, 10.1016/0014-4886(85)90117-7, 2-s2.0-0021917038. 3979505

[158] Kwo S. , Young W. , and Decrescito V. , Spinal Cord Sodium, Potassium, Calcium, and Water Concentration Changes in Rats After Graded Contusion Injury, Journal of Neurotrauma. (1989) 6, no. 1, 13–24, 10.1089/neu.1989.6.13, 2-s2.0-0024336850. 2754736

[159] Basso D. M. , Beattie M. S. , and Bresnahan J. C. , Graded Histological and Locomotor Outcomes After Spinal Cord Contusion Using the NYU Weight-Drop Device Versus Transection, Experimental Neurology. (1996) 139, no. 2, 244–256, 10.1006/exnr.1996.0098, 2-s2.0-0030157694. 8654527

[160] Kjell J. and Olson L. , Rat Models of Spinal Cord Injury: From Pathology to Potential Therapies, Disease Models & Mechanisms. (2016) 9, no. 10, 1125–1137, 10.1242/dmm.025833, 2-s2.0-84994056900. 27736748 PMC5087825

[161] Sharif-Alhoseini M. , Khormali M. , and Rezaei M. , et al.Animal Models of Spinal Cord Injury: A Systematic Review, Spinal Cord. (2017) 55, no. 8, 714–721, 10.1038/sc.2016.187, 2-s2.0-85010918085. 28117332

[162] Sroga J. M. , Jones T. B. , Kigerl K. A. , McGaughy V. M. , and Popovich P. G. , Rats and Mice Exhibit Distinct Inflammatory Reactions After Spinal Cord Injury, Journal of Comparative Neurology. (2003) 462, no. 2, 223–240, 10.1002/cne.10736, 2-s2.0-0037665153. 12794745

[163] Joshi M. and Fehlings M. G. , Development and Characterization of a Novel, Graded Model of Clip Compressive Spinal Cord Injury in the Mouse: Part 1. Clip Design, Behavioral Outcomes, and Histopathology, Journal of Neurotrauma. (2002) 19, no. 2, 175–190, 10.1089/08977150252806947, 2-s2.0-0036190467. 11893021

[164] Barber S. M. , Wolfe T. , and Steele A. G. , et al.A Novel Minimally Invasive and Versatile Kyphoplasty Balloon-Based Model of Porcine Spinal Cord Injury, Frontiers in Neurology. (2024) 15, 10.3389/fneur.2024.1422357, 1422357. 39087009 PMC11289774

[165] Robba C. , Qeva E. , Borsellino B. , Aloisio S. , Tosti G. , and Bilotta F. , Effects of Propofol or Sevoflurane Anesthesia Induction on Hemodynamics in Patients Undergoing Fiberoptic Intubation for Cervical Spine Surgery: A Randomized, Controlled, Clinical Trial, Journal of Anaesthesiology Clinical Pharmacology. (2017) 33, no. 2, 215–220, 10.4103/0970-9185.209733, 2-s2.0-85022216526. 28781448 PMC5520595

[166] Salzman S. K. , Lee W. A. , Sabato S. , Mendez A. A. , Agresta C. A. , and Kelly G. , Halothane Anesthesia Is Neuroprotective in Experimental Spinal Cord Injury: Early Hemodynamic Mechanisms of Action, Research Communications in Chemical Pathology and Pharmacology. (1993) 80, no. 1, 59–81. 8488342

[167] Ishikawa M. , Yoshitomi T. , Zorumski C. F. , and Izumi Y. , Neurosteroids Are Endogenous Neuroprotectants in an Ex Vivo Glaucoma Model, Investigative Ophthalmology & Visual Science. (2014) 55, no. 12, 8531–8541, 10.1167/iovs.14-15624, 2-s2.0-84945455364. 25406290 PMC4280088

[168] Davis J. A. and Grau J. W. , Protecting the Injured Central Nervous System: Do Anesthesia or Hypothermia Ameliorate Secondary Injury?, Experimental Neurology. (2023) 363, 10.1016/j.expneurol.2023.114349, 114349. 36775099

[169] Park H. P. , Jeon Y. T. , and Hwang J. W. , et al.Isoflurane Preconditioning Protects Motor Neurons From Spinal Cord Ischemia: Its Dose–response Effects and Activation of Mitochondrial Adenosine Triphosphate-Dependent Potassium Channel, Neuroscience Letters. (2005) 387, no. 2, 90–94, 10.1016/j.neulet.2005.06.072, 2-s2.0-23644445201. 16076524

[170] Sang H. , Cao L. , Qiu P. , Xiong L. , Wang R. , and Yan G. , Isoflurane Produces Delayed Preconditioning Against Spinal Cord Ischemic Injury via Release of Free Radicals in Rabbits, Anesthesiology. (2006) 105, no. 5, 953–960, 10.1097/00000542-200611000-00016, 2-s2.0-33750437398. 17065889

[171] Kaur J. , Gutiérrez J. F. , and Nistri A. , Neuroprotective Effect of Propofol Against Excitotoxic Injury to Locomotor Networks of the Rat Spinal Cord In Vitro, The European Journal of Neuroscience. (2016) 44, no. 7, 2418–2430, 10.1111/ejn.13353, 2-s2.0-84982206775. 27468970

[172] Yu Q. J. , Zhou Q. S. , Huang H. B. , Wang Y. L. , Tian S. F. , and Duan D. M. , Protective Effect of Ketamine on Ischemic Spinal Cord Injury in Rabbits, Annals of Vascular Surgery. (2008) 22, no. 3, 432–439, 10.1016/j.avsg.2008.03.003, 2-s2.0-42949149762. 18466821

[173] Goodman R. M. , Wachs K. , Keller S. , and Black P. , Spontaneous Spinal Cord “Injury Potential” in the Rat, Neurosurgery. (1985) 17, no. 5, 757–759, 10.1227/00006123-198511000-00005, 2-s2.0-0022394484. 2415868

[174] Nout Y. S. , Rosenzweig E. S. , and Brock J. H. , et al.Animal Models of Neurologic Disorders: A Nonhuman Primate Model of Spinal Cord Injury, Neurotherapeutics. (2012) 9, no. 2, 380–392, 10.1007/s13311-012-0114-0, 2-s2.0-84859699490. 22427157 PMC3337011

[175] Shabbir A. , Bianchetti E. , and Nistri A. , The Volatile Anesthetic Methoxyflurane Protects Motoneurons Against Excitotoxicity in an In Vitro Model of Rat Spinal Cord Injury, Neuroscience. (2015) 285, 269–280, 10.1016/j.neuroscience.2014.11.023, 2-s2.0-84916624434. 25446348

[176] Bajrektarevic D. and Nistri A. , Delayed Application of the Anesthetic Propofol Contrasts the Neurotoxic Effects of Kainate on Rat Organotypic Spinal Slice Cultures, NeuroToxicology. (2016) 54, 1–10, 10.1016/j.neuro.2016.03.001, 2-s2.0-84960079348. 26947011

[177] Aomura S. , Nakadate H. , Kaneko Y. , Nishimura A. , and Willinger R. , Stretch-Induced Functional Disorder of Axonal Transport in the Cultured Rat Cortex Neuron, Integrative Molecular Medicine. (2016) 3, no. 3, 654–660.

[178] Bottlang M. , Sommers M. B. , Lusardi T. A. , Miesch J. J. , Simon R. P. , and Xiong Z. G. , Modeling Neural Injury in Organotypic Cultures by Application of Inertia-Driven Shear Strain, Journal of Neurotrauma. (2007) 24, no. 6, 1068–1077. 17600521 10.1089/neu.2006.3772

[179] LaPlaca M. C. , Cullen D. K. , McLoughlin J. J. , and Cargill R. S.II, High Rate Shear Strain of Three-Dimensional Neural Cell Cultures: A New In Vitro Traumatic Brain Injury Model, Journal of Biomechanics. (2005) 38, no. 5, 1093–1105. 15797591 10.1016/j.jbiomech.2004.05.032

[180] Fehlings M. G. and Nashmi R. , Assessment of Axonal Dysfunction in an In Vitro Model of Acute Compressive Injury to Adult Rat Spinal Cord Axons, Brain Research. (1995) 677, no. 2, 291–299, 10.1016/0006-8993(95)00141-C, 2-s2.0-0028925713. 7552255

[181] Kouhzaei S. , Rad I. , Khodayari K. , and Mobasheri H. , The Neuroprotective Ability of Polyethylene Glycol Is Affected by Temperature in Ex Vivo Spinal Cord Injury Model, The Journal of Membrane Biology. (2013) 246, no. 8, 613–619, 10.1007/s00232-013-9574-3, 2-s2.0-84881312888. 23793797

[182] Shi R. and Blight A. R. , Compression Injury of Mammalian Spinal Cord In Vitro and the Dynamics of Action Potential Conduction Failure, Journal of Neurophysiology. (1996) 76, no. 3, 1572–1580. 8890277 10.1152/jn.1996.76.3.1572

[183] Krassioukov A. V. , Johns D. G. , and Schramm L. P. , Sensitivity of Sympathetically Correlated Spinal Interneurons, Renal Sympathetic Nerve Activity, and Arterial Pressure to Somatic and Visceral Stimuli After Chronic Spinal Injury, Journal of Neurotrauma. (2002) 19, no. 12, 1521–1529, 10.1089/089771502762300193, 2-s2.0-0036922934. 12542854

[184] Pandamooz S. , Salehi M. S. , and Zibaii M. I. , et al.Modeling Traumatic Injury in Organotypic Spinal Cord Slice Culture Obtained From Adult Rat, Tissue and Cell. (2019) 56, 90–97, 10.1016/j.tice.2019.01.002, 2-s2.0-85059936967. 30736910

[185] Weightman A. P. , Pickard M. R. , Yang Y. , and Chari D. M. , An In Vitro Spinal Cord Injury Model to Screen Neuroregenerative Materials, Biomaterials. (2014) 35, no. 12, 3756–3765, 10.1016/j.biomaterials.2014.01.022, 2-s2.0-84893754574. 24484676

[186] Takeda H. , Caiozzo V. J. , and Gardner V. O. , A Functional In Vitro Model for Studying the Cellular and Molecular Basis of Spinal Cord Injury, Spine. (1993) 18, no. 9, 1125–1133, 10.1097/00007632-199307000-00003, 2-s2.0-0027295437. 8103243

[187] Shi R. , Asano T. , Vining N. C. , and Blight A. R. , Control of Membrane Sealing in Injured Mammalian Spinal Cord Axons, Journal of Neurophysiology. (2000) 84, no. 4, 1763–1769, 10.1152/jn.2000.84.4.1763. 11024068

[188] All A. H. and Al-Nashash H. , Comparative Analysis of Functional Assessment for Contusion and Transection Models of Spinal Cord Injury, Spinal Cord. (2021) 59, no. 11, 1206–1209, 10.1038/s41393-021-00698-2. 34493803

[189] Patar A. , Dockery P. , Howard L. , and McMahon S. S. , Cell Viability in Three *ex vivo* Rat Models of Spinal Cord Injury, Journal of Anatomy. (2019) 234, no. 2, 244–251, 10.1111/joa.12909, 2-s2.0-85056352654. 30417349 PMC6326831

[190] Okada S. L. , Stivers N. S. , Stys P. K. , and Stirling D. P. , An Ex Vivo Laser-Induced Spinal Cord Injury Model to Assess Mechanisms of Axonal Degeneration in Real-Time, Journal of Visualized Experiments. (2014) 93, 10.3791/52173, 2-s2.0-84953342694, e52173. PMC4354288 25490396

[191] Petruska J. C. , Ichiyama R. M. , and Jindrich D. L. , et al.Changes in Motoneuron Properties and Synaptic Inputs Related to Step Training After Spinal Cord Transection in Rats, Journal of Neuroscience. (2007) 27, no. 16, 4460–4471, 10.1523/JNEUROSCI.2302-06.2007, 2-s2.0-34247394662. 17442831 PMC6672318

[192] Ichiyama R. M. , Broman J. , Roy R. R. , Zhong H. , Edgerton V. R. , and Havton L. A. , Locomotor Training Maintains Normal Inhibitory Influence on Both Alpha-and Gamma-Motoneurons After Neonatal Spinal Cord Transection, Journal of Neuroscience. (2011) 31, no. 1, 26–33, 10.1523/JNEUROSCI.6433-09.2011, 2-s2.0-78650881153. 21209186 PMC3036743

[193] Norreel J.-C. , Pflieger J.-F. , Pearlstein E. , Simeoni-Alias J. , Clarac F. , and Vinay L. , Reversible Disorganization of the Locomotor Pattern After Neonatal Spinal Cord Transection in the Rat, Journal of Neuroscience. (2003) 23, no. 5, 1924–1932, 10.1523/JNEUROSCI.23-05-01924.2003. 12629197 PMC6741960

[194] Jean-Xavier C. , Pflieger J.-F. , Liabeuf S. , and Vinay L. , Inhibitory Postsynaptic Potentials in Lumbar Motoneurons Remain Depolarizing After Neonatal Spinal Cord Transection in the Rat, Journal of Neurophysiology. (2006) 96, no. 5, 2274–2281, 10.1152/jn.00328.2006, 2-s2.0-33751200627. 16807348

[195] Taccola G. , Mladinic M. , and Nistri A. , Dynamics of Early Locomotor Network Dysfunction Following a Focal Lesion in an In Vitro Model of Spinal Injury, European Journal of Neuroscience. (2010) 31, no. 1, 60–78, 10.1111/j.1460-9568.2009.07040.x, 2-s2.0-72949094210. 20092556

[196] Nistri A. , Taccola G. , Mladinic M. , Margaryan G. , and Kuzhandaivel A. , Deconstructing Locomotor Networks With Experimental Injury to Define Their Membership, Annals of the New York Academy of Sciences. (2010) 1198, no. 1, 242–251, 10.1111/j.1749-6632.2009.05427.x, 2-s2.0-77952876712. 20536939

[197] Mladinic M. , Nistri A. , and Taccola G. , Aldskogius H. , Acute Spinal Cord Injury In Vitro: Insight Into Basic Mechanisms, Animal Models of Spinal Cord Repair, 2013, 76, Humana Press, 39–62, Neuromethods, 10.1007/978-1-62703-197-4_3, 2-s2.0-84875417094.

[198] Apicella R. and Taccola G. , Passive Limb Training Modulates Respiratory Rhythmic Bursts, Scientific Reports. (2023) 13, no. 1, 10.1038/s41598-023-34422-2, 7226. 37142670 PMC10160044

[199] Mohammadshirazi A. , Apicella R. , Zylberberg B. A. , Mazzone G. L. , and Taccola G. , Suprapontine Structures Modulate Brainstem and Spinal Networks, Cellular and Molecular Neurobiology. (2023) 43, no. 6, 2831–2856, 10.1007/s10571-023-01321-z. 36732488 PMC10333404

[200] Mohammadshirazi A. , Mazzone G. L. , and Zylberberg B. A. , et al.A Focal Traumatic Injury to the Spinal Cord Causes an Immediate and Massive SpReading Depolarization Sustained by Chloride Ions, With Transient Network Dysfunction and Remote Cortical Glia Changes, 2025, bioRxiv, 2024-07. 10.1007/s10571-024-01516-y PMC11695467 39745523

[201] Kojic L. , Gu Q. , Douglas R. M. , and Cynader M. S. , Serotonin Facilitates Synaptic Plasticity in Kitten Visual Cortex: An In Vitro Study, Developmental Brain Research. (1997) 101, no. 1-2, 299–304, 10.1016/S0165-3806(97)00083-7, 2-s2.0-0030879901. 9263606

[202] Jain N. , Qi H.-X. , Collins C. E. , and Kaas J. H. , Large-Scale Reorganization in the Somatosensory Cortex and Thalamus After Sensory Loss in Macaque Monkeys, Journal of Neuroscience. (2008) 28, no. 43, 11042–11060, 10.1523/JNEUROSCI.2334-08.2008, 2-s2.0-58149165232. 18945912 PMC2613515

[203] Kim B. G. , Dai H.-N. , McAtee M. , Vicini S. , and Bregman B. S. , Remodeling of Synaptic Structures in the Motor Cortex Following Spinal Cord Injury, Experimental Neurology. (2006) 198, no. 2, 401–415, 10.1016/j.expneurol.2005.12.010, 2-s2.0-33645742989. 16443221

[204] Wilson R. J. A. , Chersa T. , and Whelan P. J. , Tissue PO2 and the Effects of Hypoxia on the Generation of Locomotor-Like Activity in the In Vitro Spinal Cord of the Neonatal Mouse, Neuroscience. (2003) 117, no. 1, 183–196, 10.1016/S0306-4522(02)00831-X, 2-s2.0-0037451792. 12605904

[205] Kjaerulff O. , Barajon I. , and Kiehn O. , Sulphorhodamine-Labelled Cells in the Neonatal Rat Spinal Cord Following Chemically Induced Locomotor Activity In Vitro, Journal of Physiology. (1994) 478, no. 2, 265–273, 10.1113/jphysiol.1994.sp020248, 2-s2.0-0028142304. 7525942 PMC1155684

[206] Whelan P. , Bonnot A. , and O’Donovan M. J. , Properties of Rhythmic Activity Generated by the Isolated Spinal Cord of the Neonatal Mouse, Journal of Neurophysiology. (2000) 84, no. 6, 2821–2833, 10.1152/jn.2000.84.6.2821. 11110812

[207] Marchetti C. , Beato M. , and Nistri A. , Alternating Rhythmic Activity Induced by Dorsal Root Stimulation in the Neonatal Rat Spinal Cord In Vitro, Journal of Physiology. (2001) 530, no. 1, 105–112, 10.1111/j.1469-7793.2001.0105m.x, 2-s2.0-0034772667. 11136862 PMC2278398

